# Multiple Functions of the Type II Thioesterase Associated with the Phoslactomycin Polyketide Synthase

**DOI:** 10.1021/acs.biochem.2c00234

**Published:** 2022-11-15

**Authors:** Kyra Geyer, Steffen Hartmann, Randolph R. Singh, Tobias J. Erb

**Affiliations:** † Department of Biochemistry and Synthetic Metabolism, Max Planck Institute for Terrestrial Microbiology, Karl-von-Frisch-Street 10, D-35043 Marburg, Germany; ‡ Luxembourg Centre for Systems Biomedicine (LCSB), University of Luxembourg, Avenue du Swing 6, L-4367 Belvaux, Luxembourg; § SYNMIKRO Center for Synthetic Microbiology, Karl-von-Frisch-Street 16, D-35043 Marburg, Germany

## Abstract

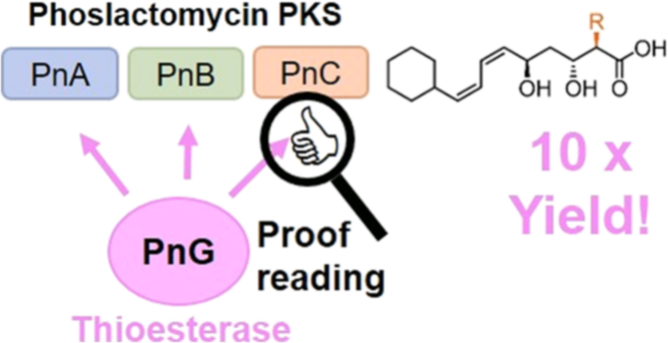

Polyketide synthases (PKSs) are molecular assembly lines that condense basic chemical building blocks for the production of structurally diverse polyketides. Many PKS biosynthetic gene clusters contain a gene encoding for a type II thioesterase (TEII). It is believed that TEIIs exert a proofreading function and restore or increase the productivity of PKSs by removing aberrant modifications on the acyl-carrier proteins (ACPs) of the PKS assembly line. Yet biochemical evidence is still sparse. Here, we investigated the function of PnG, the TEII of the phoslactomycin PKS (Pn PKS), in the context of its cognate assembly line *in vitro*. Biochemical analysis revealed that PnG preferentially hydrolyzes alkyl-ACPs over (alkyl)malonyl-ACPs by up to three orders of magnitude, supporting a proofreading role of the enzyme. We further demonstrate that PnG increases the *in vitro* production of different native and non-native tetra-, penta-, and hexaketide derivatives of phoslactomycin by more than one order of magnitude and show that these effects are caused by the initial clearing of the Pn PKS, as well as proofreading of the active assembly line. Finally, we demonstrate that PnG is able to release intermediate but notably also terminal polyketides from the Pn PKS. This allows PnG to functionally replace and overcome the terminal TEI activity of chimeric *in vitro* Pn PKS systems, as showcased with a phoslactomycin hexaketide system. Altogether, our experiments provide detailed insights into the molecular mechanisms and the multiple functions of PnG in its native context, as well as their potential use in future applications.

## Introduction

Polyketide synthases (PKSs) are multifunctional enzyme complexes that biosynthesize natural products of great structural complexity and diverse bioactivities. The structural core of these compounds is synthesized through condensation of simple (alkyl)malonyl-CoA precursors *via* the successive function of discrete PKS domains in an assembly line fashion. The final polyketide backbone is predominantly released by a covalently bound terminal thioesterase domain. Besides this terminal thioesterase, many PKSs encode a type II thioesterase (TEII). The majority of the information on TEIIs has been gained by gene disruption and replacement strategies thus far, which showed effects from complete loss of product formation, over-reduced amounts, to no change in product titers.^1−9^ Coexpression of TEIIs was successfully used to increase the yield of production,^10−12^ while strong overexpression resulted in reduced product amounts, indicating a potential tradeoff between removing blocking modifications and productive intermediates.^6,13^

It was suggested that TEIIs display an editing function and remove aberrant precursors that block (or “stall”) the assembly line, thus enabling continuous biosynthesis.^4,14^ Other reported functions of TEIIs are the release of final products,^12,15,16^ limitation and/or recycling of the CoA-thioester precursor pool,^1^ the control of starter units,^17,18^ as well as the unspecific release of short acyl residues and late-stage intermediates.^19^ The stalling of PKS assembly lines has been proposed to originate from a slow, unproductive decarboxylation of (alkyl)malonyl extender units bound to the acyl-carrier protein (ACP) during biosynthesis.^20−24^ Another potential source of unreactive alkyl-ACPs in the PKS assembly line is the conversion of *apo*-ACPs into their active *holo* form, which is catalyzed by transferases that can transfer the phosphopanthateteine (4′PP) prosthetic group from free CoA to the ACP but also acylated versions of 4′PP.^10,25,26^ The role of TEIIs in editing (*i.e.*, clearing) unreactive alkyl units is supported by data showing that these enzymes prefer decarboxylated (*i.e.*, alkyl-) over carboxylated residues and ACP-tethered over CoA/*N*-acetylcysteamine-tethered substrates.^27^

Despite their relevance, only some TEIIs have been studied *in vitro* together with the cognate NRPS or PKS system because only a few of these systems have been successfully reconstituted *in vitro* so far. YbtT, the TEII from yersiniabactin hybrid NRPS/PKS, was found to remove noncanonical precursors that blocked the synthesis *in vitro*, therefore restoring full biosynthetic activity.^13,28^ In the reconstituted type II PKS of enterocin and tetracenomycin, TEII EncL and ZhuC, respectively, were shown to impact starter unit selection.^17,18^ Besides the selection of starter units, regiospecific selection and incorporation of extender units must be controlled, especially in modular type I PKSs that incorporate different extender units, such as the phoslactomycin (Pn) PKS that uses cyclohexanecarboxyl-CoA as the starter unit and malonyl- and ethylmalonyl-CoA as extender units.

Here, we investigated the role of PnG, the TEII from the phoslactomycin biosynthetic gene cluster,^29^ in individual substrates and in the context of the assembly line using a recently established *in vitro* system.^30^ We show that PnG is a thioesterase of broad substrate tolerance that strongly prefers ACP-bound alkyl derivatives over their corresponding (alkyl-)malonyl-CoA analogues. We further show that PnG increases polyketide production with different Pn PKS systems *in vitro* through clearing and proofreading of the phoslactomycin assembly line. We finally demonstrate that PnG is capable of releasing intermediate and final polyketides and thus can replace the function of the terminal thioesterase, especially when these enzymes represent kinetic bottlenecks.

## Material and Methods

### Synthesis of Substrates

Acyl-CoA synthesis was done as previously described. cyclohexanecarboxyl-CoA was synthesized by chemical CDI coupling of the free acid, as previously described.^39^ The diketide SNAC was synthesized as previously reported.^30^

### Expression Plasmid Cloning

Preparation of the constructs for expression of PnA_V4_, PnB, PnC, and PnC-TE_DEBS_ was reported before.^30^ Please refer to the Supporting information for the respective sequences. The PnG coding sequence (optimized for expression in *Escherichia coli*) is as follows:

ATGGTGACCGAAAGCGATCGCTGGATTCGCAGCTATCTGCCGGGCCCGGCGGAAGCGCTGCCGGTGGTGCATTTTCCGCATGCGGGCGGCAGCGCGAGCTATTATCGCCCGTTTTGCGCGGCGCTGAGCGATCGCTTTAACGCGCTGGCGCTGCAGTATCCGGGCCGCCAGGATCGCCGCGATGAACCGTGCGTGACCGATCTGCATGTGCTGGCGGATCTGCTGTTTGATCGCCTGCGCCAGTGCGCGGATCGCCCGATGGCGTTTTTTGGCCATAGCATGGGCGCGCTGCTGGCGTTTGAAGTGACCCGCCGCTTTGAACGCGAACTGAACACCAGCCCGGTGGCGCTGTTTCTGAGCGGCCGCCGCGCGCCGAGCCGCCATCGCGATGAAAACGTGGATCTGAGCAGCAACGAAAGCCTGCTGGCGGAAATTCGCGAACTGAGCGGCACCGATCCGCGCCTGCTGGGCGATGATGAAATGCTGGAAATGATTATGGAACCGCTGCGCGCGGATTATCAGGCGCTGGGCGCGTATCATTTTGCGCCGGAACCGCCGGTGCGCTGCCCGGTGACCGTGCTGACCGGCGCGGATGATCCGCGCACCAGCCAGGATGAAGCGGCGGCGTGGCAGGAACATACCACCGGCGCGTTTGATCTGCGCGTGTTTCCGGGCGGCCATTTTTTTATTAGCGAAAACGTGGCGGATGTGACCGGCTTTGTGGCGGAACGCCTGAGCGCGGTGCCGCTGGCGGGCTAA

The PnG coding sequence was inserted between the *Nde*I and *Hin*dIII multiple cloning sites of pET28b(+). The ordered sequence contained 5′ and 3′ overhangs of 8 bp length to enable restriction by *Nde*I and *Hin*dII. Plasmid pET28b(+) was digested with the same overhangs, and the PnG and linearized pet28b(+) were purified from 0.8% agarose gel and ligated by a T4 ligase-mediated reaction. Single colonies were picked and grown, and the plasmid was isolated and sequenced.

### Protein Production and Nickel NTA Purification

Electrocompetent *E. coli* BAP1 cells were transformed with expression constructs encoding for Pn PKS; for expression of all other proteins used in this study, including standalone ACP domains, *E. coli* BL21 (DE3) competent cells were transformed with the respective expression constructs and supplemented with the respective antibiotics. For PnG, 1 L of Terrific Broth medium was inoculated from the plate and grown at 37 °C until the OD_600_ reached 1.8. The culture was placed shaking at 23 °C, and expression was induced with 100 μM isopropyl-β-d-thiogalactopyranoside. After 4 h of incubation, the culture was harvested and used for protein purification or stored at −20 °C. For all other expressions, a 10 mL overnight culture was grown and used to inoculate 1 L of Terrific Broth medium. The cultures were grown at 37 °C until an OD_600_ of 1 was reached and then placed shaking at 18 °C (Pn PKS proteins) or 20 °C overnight. The cultures were harvested and used for protein purification or stored at −20 °C. Cell pellets were resuspended in buffer A (500 mM NaCl, 50 mM NaH_2_PO_4_, 10% v/v glycerol, pH 7.5), lysed by sonication, and centrifuged at 42,000*g* and 4 °C for 45 min, and the supernatant was mixed with 3.5 mL of Protino Ni-NTA agarose purchased from Macherey Nagel and incubated for 2 h on ice with slow shaking. The bead solution was transferred into Protino Columns 14 mL and washed with 50 mL of buffer A. Afterward, the beads were washed with 20 mL of washing buffer (25 mM imidazole, 500 mM NaCl, 50 mM NaH_2_PO_4_, 10% v/v glycerol, pH 7.5). Proteins were eluted with 8 mL of buffer B (500 mM imidazole, 500 mM NaCl, 50 mM NaH_2_PO_4_, 10% v/v glycerol, pH 7.5). PnG and all standalone ACP proteins were further purified by size-exclusion using a HiLoad 16/600 Superdex 200 pg column (300 mM NaCl, 25 mM NaH_2_PO_4_, pH 7.5). Pn PKS proteins were subjected to anion exchange. Protein concentration was determined by UV–vis measurements at 280 nm; in the case of ACPs, the concentration was determined by the Bradford assay.

### Anion Exchange

The eluate from the nickel bead purification was diluted to 120 mL with anion A buffer (50 mM NaH2PO4, pH 7.5) and loaded onto a 5 mL HiTrap Q HP anion exchange 5 mL chromatography column, purchased from GE Healthcare Life Sciences, with a flow of 3 mL min^–1^. A gradient to 100% anion B buffer (50 mM NaH_2_PO_4_, 500 mM NaCl pH 7.5) with a flow of 4 mL min^–1^ was run over 30 min, and protein-containing fractions were collected and concentrated using Amicon Ultra Centrifugal Filters, purchased from Merck Millipore. Proteins were used immediately for assays or stored in 30% v/v glycerol at −80 °C after shock freezing in liquid nitrogen.

### Pn PKS and PnG *In Vitro* Assays

The production of phoslactomycin polyketide derivatives was initiated using PnA_V4_ and cyclohexanecarboxyl-CoA. The assay was run at a volume of 50 μL and contained 5 μM Pn PKS proteins, 3.5 μM Npt, 1 mM cyclohexanecarboxyl-CoA, 1 mM malonyl-CoA and 0.5 mM α-substituted malonyl-CoA derivatives (in single extender unit assays, extender units were provided at 1 mM concentration), 5 mM NADPH, 0.1 mM CoA, and 5 mM MgCl_2_. The reaction was buffered with 100 mM NaH_2_PO_4_. PnG concentrations used in the assay varied and were adjusted to the ACPs present in the assay solution. PnG relative concentrations were 0.1, 0.25, 0.5, 1, and 2, whereas a PnG concentration of 1 equaled the molar concentration of ACPs in the whole assembly line and 0.1 equaled a tenth of the ACPs present. When bypassing PnA_V4_, (2*Z*)-cyclohexanepropenyl-SNAC^4^ was used as the diketide substrate analogue. Immediately after mixing the reaction, 10 μL was added to 10 μL of methanol as the negative control. The assay was run at 25 °C, and samples were taken after 2 h and overnight and quenched 1:1 with methanol.

### Polyketide Production Assay with Pretreated Pn PKS Enzymes

Phoslactomycin production assays to study the effect of TEII on continuous PnG activity were performed as follows. To assure that the Pn PKS proteins did not contain any misloaded acyl residues, the ACPs had to be cleared during protein preparation. For this, PnA_V4_, PnB, and PnC-TE_DEBS_ were expressed as described above. After Ni-NTA purification, the proteins (including PnG) were desalted using a PD-10 column and concentrated to a volume of approximately 1 mL. Each Pn PKS enzyme was treated separately with 5 μM Npt, 1 mM CoA, 5 μM PnG for 45 min in 10 mM MgCl_2_, and 100 mM NaH_2_PO_4_ at 28 °C (assures complete activation of the ACPs to form *holo*-ACPs and complete removal of eventual acyl residues bound to the ACPs). After this treatment, the Pn PKS enzymes were cleared of PnG and CoA by size-exclusion chromatography on a HiLoad 16/600 Superdex 200 pg column (300 mM NaCl, 25 mM NaH_2_PO_4_, pH 7.5). The assay was run at a volume of 50 μL and contained 5 μM Pn PKS proteins, 1 mM cyclohexanecarboxyl-CoA, 1 mM malonyl-CoA, 0.5 mM each α-substituted malonyl-CoA derivatives, 5 mM NADPH, and 100 mM NaH_2_PO_4_. PnG concentrations used in the assay varied and were adjusted to the number of ACPs present in the assay solution. PnG was used at relative concentrations of 0.1, 0.25, 0.5, 1, and 2, whereas a relative PnG concentration of 1 equaling the molar concentration of ACPs was used for the whole assembly line. Immediately after mixing the reaction, 10 μL was added to 10 μL of methanol as the negative control. The assay was run at 25 °C, and samples were taken after 2 h and overnight and quenched 1:1 with methanol.

### High-Resolution Mass Spectrometry Analysis of the Polyketide Production Assay

All samples were measured immediately or stored at −80 °C. UPLC–high-resolution MS analysis was carried out using an Agilent 6550 iFunnel QTOF LC-MS system equipped with an electrospray ionization source set to positive ionization mode. The analyte was separated on an RP-18 column (50 mm × 2.1 mm, particle size 1.7 μm, Kinetex EVO C18, Phenomenex) using a mobile phase system comprising 0.1% formic acid in water (solvent A) and acetonitrile (solvent B). Chromatographic separation was carried out using the following gradient conditions at a flow rate of 250 μL min^–1^: 0 min 5% B; 1 min 5% B, 6 min 95% B; 6.5 min 95% B; 7 min 5% B. The column oven was set to 40 °C, and the autosampler was maintained at 8 °C. The standard injection volume was 10 μL. The capillary voltage was set at 3.5 kV, and nitrogen gas was used as nebulizing (20 psig), drying (13 L min^–1^, 225 °C), and sheath (12 L min^–1^, 40 °C) gas. MS data were acquired in the scan range of 50–1200 *m*/*z*. LC–MS data were analyzed using MassHunter Qualitative Analysis software (Agilent).

### Tandem Mass Spectrometry Analysis of Pentaketides

Analysis of polyketides was performed using HRES–LC–MS. The chromatographic separation was performed on a Thermo Scientific Vanquish HPLC System using a Kinetex Evo C18 column (50 mm × 0.12 mm, 100 A, 1.7 μm, Phenomenex) equipped with a 20 mm × 2.1 mm guard column of similar specificity at a constant eluent flow rate of 0.25 mL min^–1^ and a column temperature of 40 °C with eluent A being 0.1% formic acid in water and eluent B being 0.1% formic acid in acetonitrile (Honeywell). The injection volume was 2 μL. The elution profile consisted of the following steps and linear gradients: 0–2 min constant at 0% B; 2–11 min from 0 to 100% B; 11–12 min constant at 100% B; 12–13 min from 100 to 0% B; 13–14 min constant at 0% B. A Thermo Scientific ID-X Orbitrap mass spectrometer was used in positive mode with an electrospray ionization source under the following conditions: ESI spray voltage 5000 V, sheath gas at 45 arbitrary units, auxiliary gas at 9 arbitrary units, sweep gas at 7 arbitrary units, ion transfer tube temperature at 300 °C, and vaporizer temperature at 325 °C. Scheduled targeted collision-induced dissociation was performed on the five suspect molecules, applying a precursor ion scan at a mass range between 200 and 400 *m*/*z* with a mass resolution of 120 000 using the Orbitrap mass analyzer after quadrupole pre-isolation. Data-dependent detection of MS2 spectra was performed at a normalized collision energy of 30% and an activation time of 10 ms with an automatic definition of the scan range and a mass resolution (MS2) of 120 000 using the Orbitrap mass analyzer.

### Kinetic Analysis of PnG

ACP loading reactions contained 2 mM ACP, 10 μM Npt, 5 mM MgCl_2_, and 2.4 mM acyl-CoA (except for butyl-CoA, where 1.5 mM ACP and 1.6 mM butyl-CoA were used). After incubation of the loading reaction for 2 h at 28 °C, the reaction was stored on ice. For the kinetic characterization of PnG, mixtures with individual concentrations of acyl-ACP (end concentrations between 1.8 mM and 5 μM) were treated with PnG (end concentration 20 nM in case of decarboxylated alkyl-ACPs and 500 nM in case of carboxylated acyl-ACPs) for individual durations between 1 and 20 min in a total volume of 10 μL. Subsequently, reactions were quenched with 10 μL of 20% (v/v) formic acid. The quenched reaction solutions were diluted with water to a final ACP concentration of 10 μM. Two microliters of the buffered protein solutions was desalted online using a Waters ACQUITY H-Class HPLC system equipped with a MassPrep column (Waters). Desalted proteins were eluted into the ESI source of a Synapt G2Si mass spectrometer (waters) by the following gradient of buffer A (water with 0.05% formic acid) and buffer B (acetonitrile with 0.045% formic acid) at a column temperature of 60 °C and a flow rate of 0.1 mL min^–1^: isocratic elution with 5% A for 2 min, followed by a linear gradient to 95% B within 8 min, and holding 95% B for additional 4 min. Positive ions within the mass range of 500–5000 *m*/*z* were detected. Glu-fibrinopeptide B was measured every 45 s for automatic mass drift correction. Averaged spectra were deconvoluted after baseline subtraction and eventually smoothed using MassLynx instrument software with MaxEnt1 extension.

### PnG Hydrolysis of CoA-Thioesters

To assure that PnG does not hydrolyze polyketide substrates, an ultrahigh-performance liquid chromatography supported assay was performed. For this, 5 μM PnG was incubated with 1 mM malonyl-, methylmalonyl-, ethylmalonyl-, butylmalonyl-, 3-methylbutylmalonyl-, hexylmalonyl-, or cyclohexanecarboxyl-CoA in 50 mM NaH_2_PO_4_, pH 7.5 at 25 °C. The control to test for background hydrolysis of the CoA esters did not contain PnG. Samples were taken at 0, 60, and 240 min and quenched with formic acid (final in sample 10% v/v). The samples containing malonyl-, methylmalonyl-, and ethylmalonyl-CoA were separated on a Eurospher II 100-2 C18 column (100 mm × 2 mm, Knauer). Separation of these CoA-thioesters in the reaction samples was done with a gradient of 1.5–10% (v/v) acetonitrile in 10 mM potassium phosphate buffer (pH 6.8) over 6.5 min at a flow rate of 0.2 mL min^–1^ at 50 °C. The samples containing cyclohexanecarboxyl-, butylmalonyl-, 3-methylbutylmalonyl-, and hexylmalonyl-CoA were separated on a Luna 100-2 C18 column (150 mm × 4.6 mm, Phenomenex). Separation of these CoA-thioesters in the reaction samples was done with a gradient of 2–18% (v/v) acetonitrile in 25 mM ammoniumformiate buffer (pH 8.1) over 9 min at a flow rate of 0.25 mL min^–1^ at 30 °C. To detect the CoA-thioesters *via* UV absorbance at 260 nm, an InfinityLab Max-Light cartridge cell was used (60 mm detector length, Agilent Technologies Inc., Santa Clara).

## Results and Discussion

### Heterologous Expression and Purification of PnG and Pn PKS Enzymes

The 6xHis-tagged PnG protein was purified to homogeneity by Ni-NTA purification, followed by size-exclusion chromatography (Figure S1). According to size-exclusion chromatography, PnG eluted as a monomeric protein of 34.4 kDa size (calculated 30.17 kDa), which is in line with the assumption that TEIIs function as monomers.^27,31,32^ Active-site knockout PnG S93G showed the same elution patterns as the wild-type enzyme. Enzymes of the Pn PKS were purified as previously described.^30^

### PnG Preferentially Hydrolyses Alkyl-ACPs

First, we determined the kinetic parameters of PnG with different ACP-bound acyl substrates. For this, we used two different ACPs from the phoslactomycin PKS (Pn PKS), ACP_LD_ (from the loading module of PnA) and ACP2 (from the first module of PnB), as substrate templates and loaded them with different acyl residues using Npt, the promiscuous phosphopantetheinyl-transferase from *Streptomyces platensis*.^30^

Overall, PnG was equally active with both ACPs (Table 1 and Figure S2), which is in line with the observation that TEIIs generally show low specificity toward the carrier protein.^2,6,13^ Our kinetic analysis showed distinct differences in the hydrolytic activity of PnG between alkyl- and (alkyl)malonyl-CoA substrates. PnG hydrolyzed all tested alkyl-ACPs (*i.e.*, acetyl-, propionyl-, and butyl-ACP) with comparable specificity constants ranging from 2.2 × 10^5^ to 6 × 10^5^ M^–1^ s^–1^, indicating no specificity for alkyl chain length as observed earlier for RedJ, the TEII in the prodiginine biosynthesis cluster.^31^ On the other hand, when testing (alkyl)malonyl-ACP substrates (*i.e.*, malonyl-, methylmalonyl-, and ethylmalonyl-ACP), we observed a 45–50-fold preference for malonyl- over methyl- and ethylmalonyl-ACP, indicating that PnG effectively discriminates between shorter and longer (alkyl)malonyl-ACP substrates, discriminating against the natural substrate ethylmalonyl-ACP.

**Table 1:** Reaction Parameters for PnG Hydrolytic Activity with Various Acyl Residues Bound to ACP_LD_ and ACP2 with the Standard Errors

substrate	*k*_cat_ [min^–1^]	*K*_M_ [μM]	*k*_cat_/*K*_M_ [M^–1^ s^–1^]
Decarboxylated Substrates			
**acetyl-ACP**_**LD**_	618 ± 45	47 ± 17	2.2 × 10^5^ ± 8 × 10^4^
**butyl-ACP**_**LD**_	493 ± 38	23 ± 9	3.6 × 10^5^ ± 1.4 × 10^5^
**acetyl-ACP2**	965 ± 56	27 ± 7	6 × 10^5^ ± 1.6 × 10^5^
**propionyl-ACP2**	2050 ± 358	139 ± 57	2.5 × 10^5^ ± 1.1 × 10^5^
**butyl-ACP2**	353 ± 39	23 ± 12	2.6 × 10^5^ ± 1.4 × 10^5^
Carboxylated Substrates			
**malonyl- ACP**_**LD**_	259 ± 31	293 ± 109	1.5 × 10^4^ ± 5.8 × 10^3^
**ethylmalonyl- ACP**_**LD**_	10 ± 0.8	501 ± 113	3.3 × 10^2^ ± 8 × 10^1^
**malonyl-ACP2**	77 ± 13	164 ± 99	7.8 × 10^3^ ± 4.9 × 10^3^
**methylmalonyl-ACP2**	20 ± 5	369 ± 196	9 × 10^2^ ± 5.3 × 10^2^
**ethylmalonyl-ACP2**	2 ± 0.3	168 ± 72	2 × 10^2^ ± 9 × 10^1^

When directly comparing hydrolytic activity between the different (alkyl)malonyl-ACP substrates and their corresponding “decarboxylated” analogues, we observed that PnG displays a strong preference for the latter, which is in line with the hypothesis that PnG functions as a proofreading enzyme, which recycles “decarboxylated” ACPs that cannot undergo Claisen condensation and thus represent dead-end intermediates. PnG preferred acetyl-ACP over malonyl-ACP with a 14–75-fold increased specificity constant. In cases of propionyl-/methylmalonyl-ACP and butyl-/ethylmalonyl-ACP, we determined 150-fold and 1000–1500-fold increases in specificity, respectively. Preferences for decarboxylated over carboxylated substrates were also reported with other TEIIs;^5,13,27,31^ however, they were not as pronounced as in the case of PnG. Our kinetic data showed that the preference for the decarboxylated substrate is mainly caused by increased turnover rates and additionally decreased K_M_ values against the different alkyl-ACP substrates compared to their (alkyl-)malonyl-ACP analogues. Altogether, these results suggest that PnG effectively hydrolyzes alkyl-ACPs, while the hydrolysis of carboxylated substrates from ACPs is negligible, indicating that PnG is able to specifically proofread decarboxylated ACP substrates stalling on the assembly line.

### PnG Increases Productivity of the Pn Assembly Line *In Vitro*

**Scheme 1:**
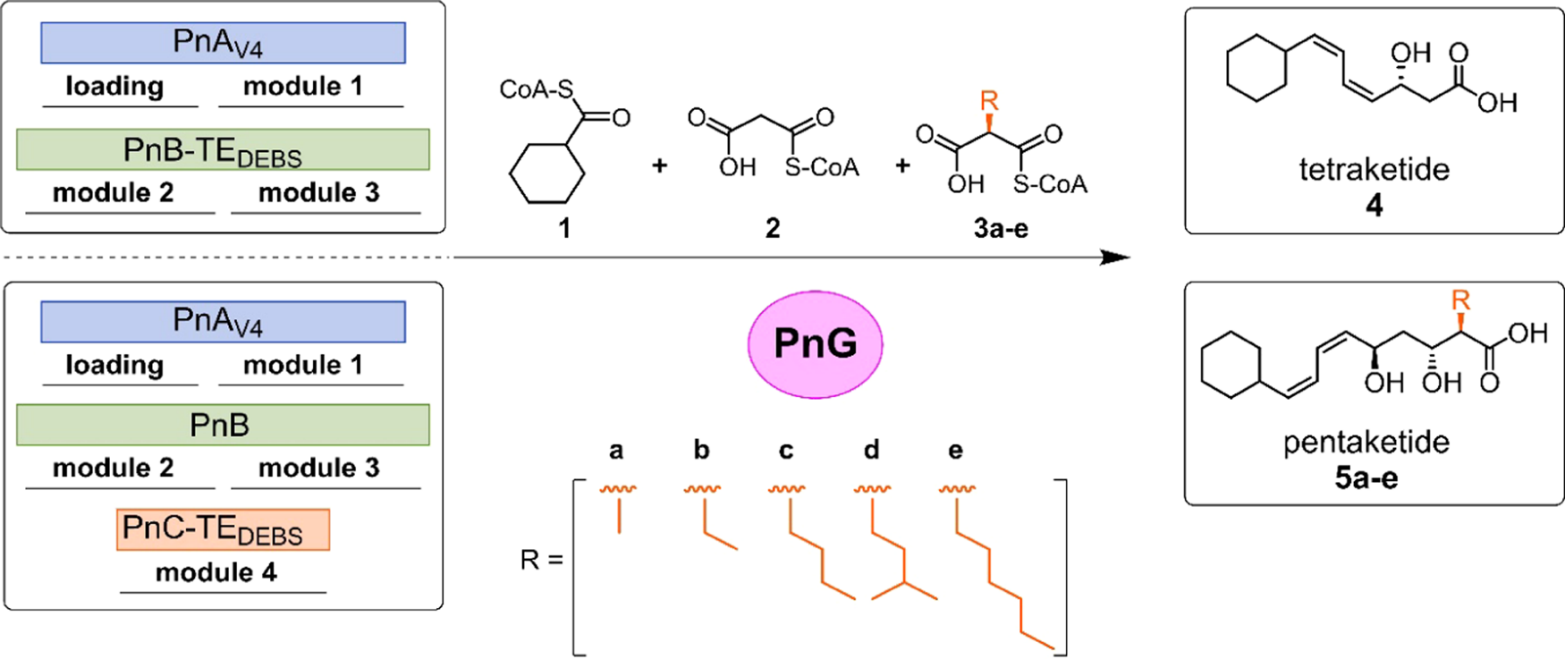
Assay Scheme for Characterization of PnG Tetraketide (**4**) or pentaketide (**5**) production is initiated by PnA_V4_ and terminates with PnB-TE_DEBS_ in the case of **4** or with PnC-TE_DEBS_ in the case of **5**. Substrates provided are cyclohexanecarboxyl-CoA (**1**), malonyl-CoA (**2**) and in standard pentaketide production assays one of the following α-substituted extender units, in the case of competitive assays, all five α-substituted extender units (**a**, methyl-; **b**, ethyl-; **c**, butyl-; **d**, 3-methylbutyl-; and/or **e**, hexylmalonyl-CoA). Ethylmalonyl-CoA **3b** is the native substrate of PnC; however, **3a**–**3e** are incorporated into the pentaketide. For the found masses, please refer to Figure S4 and Table S1.

To investigate whether PnG indeed directly affects phoslactomycin biosynthesis, we tested the enzyme in the context of a recently established Pn PKS *in vitro* system for the production of tetra- and pentaketides (Scheme 1).^30^ In this system, polyketide biosynthesis is initiated by PnA_V4_ with cyclohexanecarboxyl-CoA and malonyl-CoA and continued by PnB using two malonyl-CoA extender units, yielding the tetraketide (**4**). For production of the pentaketide (**5**), another round of extension of **4** is followed by PnC. While PnC incorporates ethylmalonyl-CoA (**3b**) in the native context (Figure S3), its acyltransferase (AT) is promiscuous and also accepts different non-native extender units (**3a**, **3c**–**3e**), which leads to pentaketides **5a**–**5e** when these non-native extenders are provided.

**Figure 1:**
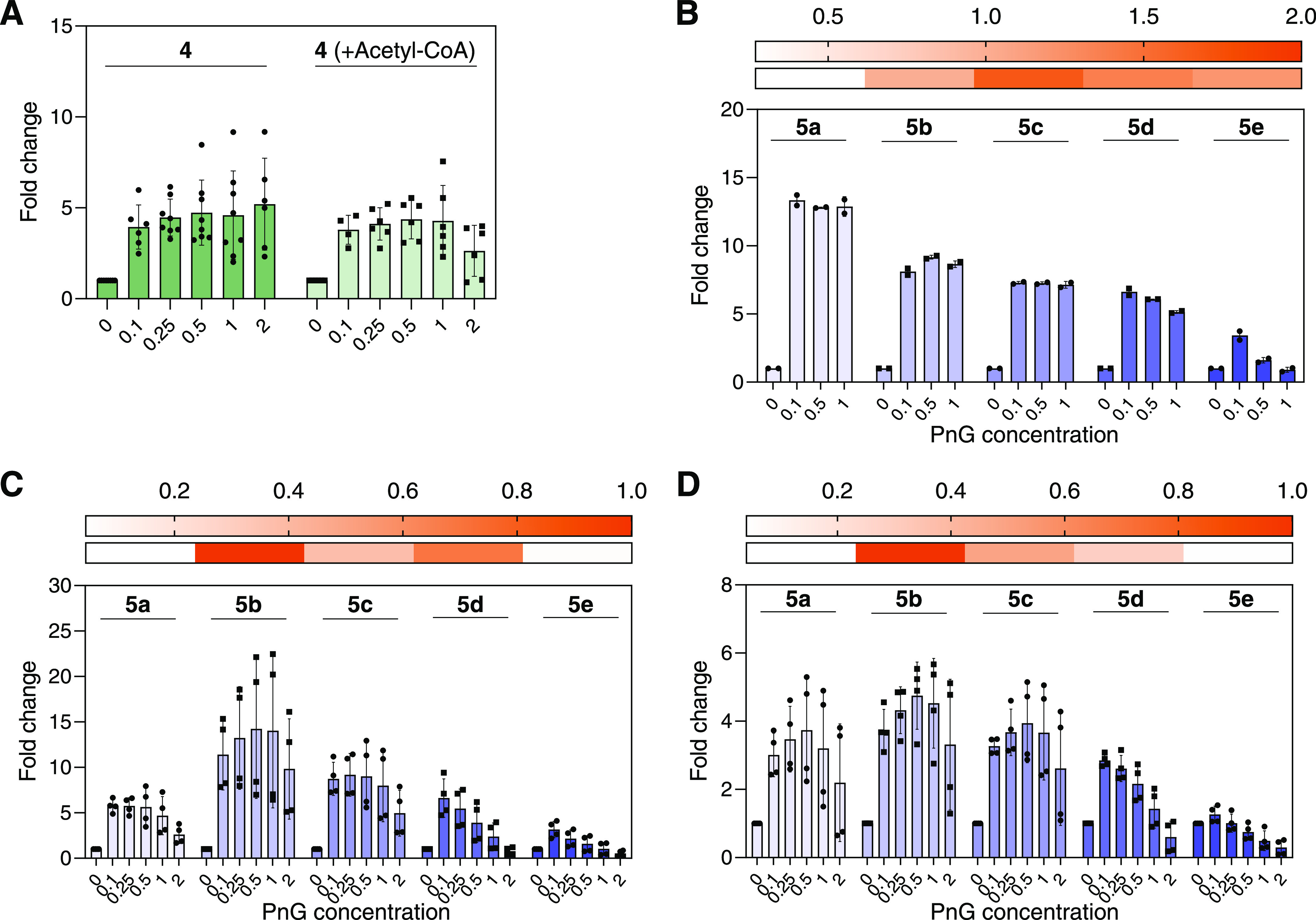
Effect of increasing PnG concentration on polyketide production *in vitro*. Concentrations of PnG ranged between 0.1 and 2 molar equiv of ACPs present in the assay, indicated on the *x*-axis. All values were set relative to the control without PnG. Samples were taken after 2 h and measured by mass spectrometry. (A) Tetraketide **4** production with the native substrates and additionally with acetyl-CoA. (B) Pentaketide **5a**–**e** production with **2** and one α-substituted malonyl-CoA derivative **3a**–**e** as extending substrates. The heatmap shows the initial relative amounts of pentaketides **5a**–**e** in the absence of PnG, set relative to native pentaketide **5b**. (C) Pentaketide production in competitive conditions with all substrates **1**, **2**, and **3a**–**e**. The heatmap shows the initial relative amounts of pentaketides **5a**–**e** in the absence of PnG, set relative to native pentaketide **5b**. (D) Pentaketide **5**–**e** production in competitive conditions with PnG-pretreated Pn PKS enzymes. Preincubation with PnG assures removal of all acyl residues. The heatmap shows the initial relative amounts of pentaketides **5a**–**e** in the absence of PnG, set relative to native pentaketide **5b**. Residues indicated are as follows: **a** = methyl-, **b** = ethyl-, **c** = butyl-, **d** = 3-methlbutyl-, and **e** = hexyl-residues. A negative control with PnG S93G catalytic knockout was used (Figure S6). Data points are from multiple biological (A, C, D) and technical (A–D) replicates. For a list of the product masses, refer to Table S1.

When adding PnG to the tetraketide system, the production of **4** increased between 4- and 5-fold, independent of the PnG concentration (Figures 1A and S5). Addition of PnG S93G, a catalytic knockout control, did not impact polyketide biosynthesis (Figure S6), supporting the hypothesis that PnG increases product formation by proofreading stalled assembly lines. To test whether misloading of ACP_LD_ (the first step in Pn biosynthesis) might cause assembly line stalling, we tested the effect of acetyl-CoA, a common metabolite that is present at millimolar concentrations in the cell, on tetraketide production. When challenging tetraketide assays by adding 1 mM acetyl-CoA, production of **4** was unaffected by the presence or absence of PnG (Figure 1A), which ruled out acetyl-CoA misloading of ACP_LD_ as a major factor in assembly line stalling (Figure 1A). This was further supported by the fact that no production of modified **4** as a result of acetate starter incorporation was observed in the presence or absence of PnG. Altogether these experiments strongly suggested that PnG acts as a proofreading enzyme on the active phoslactomycin assembly line, increasing product formation.

Next, we tested the effect of PnG on the pentaketide system (Scheme 1). For the pentaketide system, we supplied **1**, **2**, and either one α-substituted extender unit (**3a**, **3b**, **3c**, **3d**, or **3e**) alone or all five α-substituted extender units (**3a**–**3e**) at the same time in a competitive assay. With these assays, we aimed to test the effects of PnG on phoslactomycin biosynthesis in the presence of native (**2** and **3b**) and individual non-native (**3a**, **3c**, **3d**, or **3e**) extender units (Figure 1B) and the effects of PnG on an assembly line that is challenged with multiple substrates simultaneously (Figure 1C).

In pentaketide assays in the absence of PnG, we detected pentaketide distributions of **5a**–**5e** as reported before, with minor amounts of **5a** formed and with **5b**–**d** as the main products^30^ (Figures 1B,C and S5). Tandem mass spectrometry confirmed the structures of pentaketides **5a**–**5e** (Figure S7). Similar to the tetraketide system, upon the addition of PnG, product formation strongly increased in the pentaketide system. In assays with only one single α-substituted extender unit added (Figure 1B), formation of **5a** was increased 13-fold (the initial amount had been close to the detection limit), while **5b**–**d** were increased around 9-fold and production of **5e** was improved only 3-fold, remaining unaltered at high concentrations of PnG.

In competitive extender unit assays containing all five α-substituted extender units (Figures 1C and S5), we observed an increase of the production of **5a**–**5e**, especially at low concentrations of PnG. The beneficial effect of PnG was most pronounced on **5b**, the native product, with a 14-fold increase, followed by **5c** (9-fold), **5d** (7-fold), **5a** (6-fold), and **5e** (3-fold). Overall, these results showed that PnG generally has a beneficial effect on tetra- and pentaketide production *in vitro*, with product increases of up to 14-fold upon addition of PnG.

### PnG Acts Directly on the Active Pn Assembly Line

As mentioned above, TEIIs remove unwanted modifications on the reactive thiol group of the 4′-PP cofactor of ACPs that block the assembly line. These modifications can originate from the slow, unproductive decarboxylation of (alkyl)malonyl-ACPs or the misloading of ACPs by promiscuous phosphopantetheinyl-transferases. To test whether misloading of ACPs during protein production in *E. coli* BAP1 played a role, we pretreated purified Pn PKS enzymes with the native transferase from *S. platensis* Npt, CoA, and PnG to ensure complete activation and clearing of potential acyl residues from ACPs during protein expression. PnG and Npt were removed by size-exclusion chromatography and Npt was omitted from the assays to circumvent misloading of any residual *apo*-ACPs. Notably, even for these pretreated enzymes, an increase of pentaketide production could still be observed upon addition of PnG, although at reduced levels. In pretreated enzymes, production of **5a**, **5b**, **5c**, and **5d** was increased between 3- and 5-fold, while the effect on **5e** was negligible (Figures 1D and S5). Overall, these experiments suggested that the action of PnG in the *in vitro* system is a cumulative effect of clearing misloaded ACPs before, but notably also proofreading during phoslactomycin biosynthesis, when slow unproductive decarboxylation of (alkyl)malonyl-ACPs takes place in the active Pn PKS assembly line. Further efforts to identify specific proofreading targets of PnG in the active assembly line with SNAC feeding studies proved unfortunately unsuccessful, as SNAC esters apparently inhibited PnG (Figure S8).

In general, low concentrations of PnG (*i.e.*, around or below 0.5 molar ratio) showed the best effects, resulting in the highest product titers for native and non-native substrates (Figure 1D), while high PnG concentrations (*i.e.*, above 1 molar ratio) did stimulate less product formation (Figure 1D, **5a**–**5c**) or even lowered product formation, especially in the case of non-native products **5d** and **5e** with branched and long-chain moieties, respectively (Figure 1D). This notably shaped the product profile in competitive pentaketide assays toward formation of the native product (**5b**) (Figures 1D and S10).

The effects observed at high PnG concentrations could be caused by increased PnG-catalyzed clearing and/or proofreading of ACP-bound non-native (alkyl)malonyl extender units or hydrolysis (and thus depletion) of the corresponding CoA esters in the assay, as some TEIIs were shown to hydrolyze CoA-thioesters with low activities.^26,27^ To test the latter hypothesis, we incubated PnG with all CoA-thioester substrates. Even after several hours of incubation, no significant CoA ester hydrolysis was observed (Figure S9). Thus, we concluded that the decreased production levels of non-native pentaketides at high PnG concentrations were mainly due to the increased hydrolysis of ACP-bound (alkyl)malonyl extenders. This was probably caused by slower turnover and thus increased exposition of ACP-bound non-native extender units toward PnG.

### Release of Polyketides by PnG

**Figure 2:**
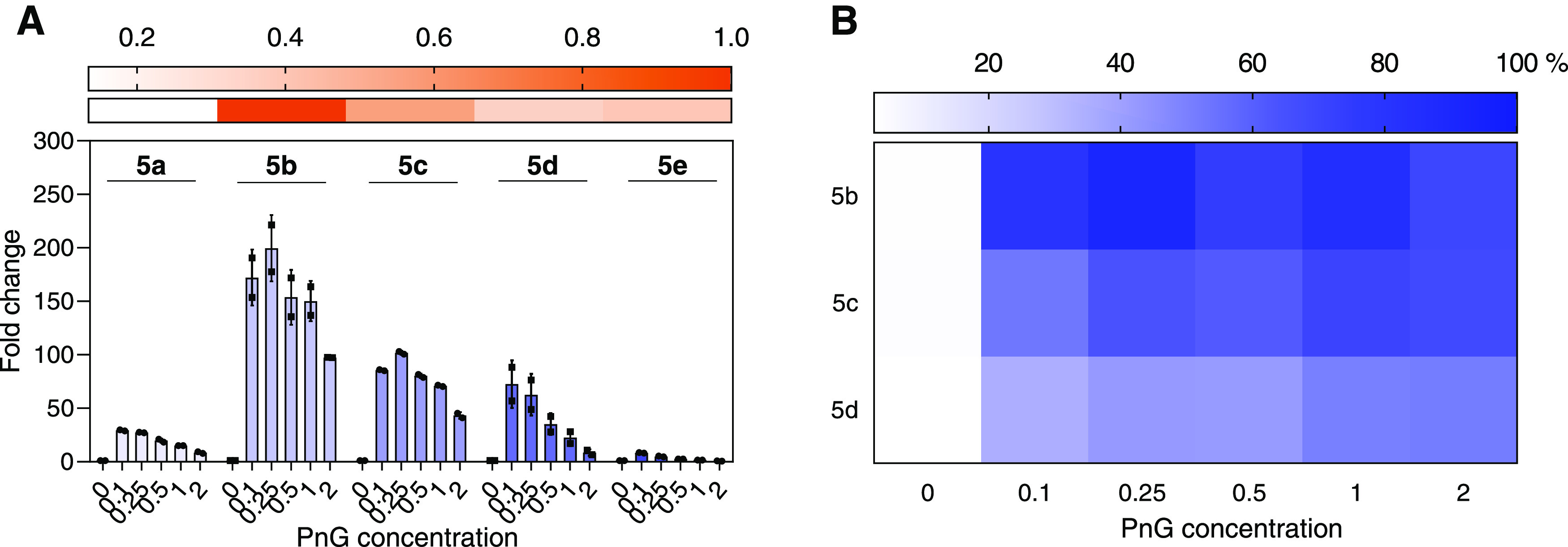
Pentaketide production in competitive assays lacking a terminal thioesterase. Concentrations of PnG ranged between 0.1 and 2 molar equivalents of ACPs present in the assay, indicated on the x-axis. All samples were taken after 2 h incubation and analyzed by mass spectrometry. (A) Heatmap displaying the initial relative amounts of the pentaketides **5a**–**e** in the absence of PnG, set relative to native pentaketide **5b**. A strong increase of product formation upon addition of PnG can be observed for all pentaketides, in particular **5b**–**5d**. (B) Heatmap showing the production yield in the percentage of assays terminating without TE_DEBS_ (PnA_V4_, PnB, PnC) compared to assays terminating with TE_DEBS_ (PnA_V4_, PnB, PnC-TE_DEBS_) with increasing PnG concentrations. Results are shown for the most prominent products **5b**, **5c**, and **5d**. Residues indicated are as follows: **a** = methyl-, **b** = ethyl-, **c** = butyl-, **d** = 3-methlbutyl-, and **e** = hexyl-residues.

Finally, we assessed whether PnG does not only release ACP-bound extender units but would also be able to hydrolyze polyketides from the assembly line. Therefore, we first tested the pentaketide assembly line in a competitive assay without the terminal thioesterase TE_DEBS_ (PnA_V4_ + PnB + PnC) (Figures 2 and S11). Without TE_DEBS_, the initial product distribution pattern was similar to assays terminating with TE_DEBS_. However, product yields were strongly reduced (in all cases below 5%), verifying the relevance of TE_DEBS_ as a terminating domain in the *in vitro* pentaketide system. Addition of PnG in low to medium concentrations to the system without TE_DEBS_ restored product formation in all cases tested. Production of **5a** and **5e** was increased by about one order of magnitude compared to the system without the DEBS thioesterase, while formation of **5c** and **5d** was increased by about two orders of magnitude, and formation of the native product **5b** was increased by 200-fold. Compared to the system terminating with TE_DEBS_, final product levels reached 50% (**5d**), 70% (**5c**), and 90% (**5b**) with PnG, demonstrating that PnG is able to replace the thioesterase function of TE_DEBS_ in the pentaketide *in vitro* system.

**Figure 3:**
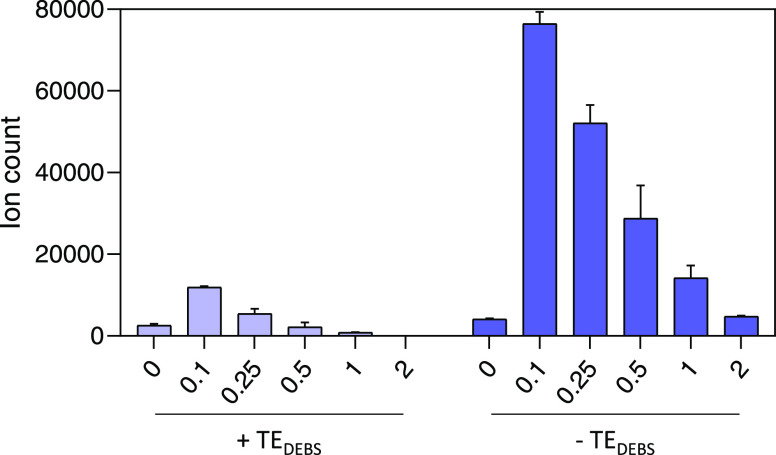
Release of hexaketides by PnG. The *x*-axis shows the concentration of PnG, equimolar to ACPs present in the assay. On the left side, PnG has been added to assays terminating with TE_DEBS_ (PnA_V4_, PnB, PnC, PnD-TE_DEBS_), and on the right side, PnG has been added to assays terminating without TE_DEBS_ (PnA_V4_, PnB, PnC, PnD). A 5-fold increase in product formation can be observed upon addition of PnG to assays terminating with TE_DEBS_, while a 20-fold increase can be observed in the absence of TE_DEBS_. In the control samples without PnG, assays terminating without TE_DEBS_ contain 2-fold higher product amounts than when a terminal TE is present. This shows a negative influence of the terminal TE from DEBS on Pn hexaketide production.

We also tested whether PnG would be able to replace TE_DEBS_ in a phoslactomycin hexaketide system (PnA_V4_ + PnB + PnC + PnD).^30^ Unexpectedly, hexaketide yields increased 2-fold in the absence of TE_DEBS_, indicating that TE_DEBS_ activity is nonfunctional in the hexaketide system and apparently even inhibits hexaketide release. Addition of PnG increased product formation by 20-fold for the system without TE_DEBS_ and (still) 5-fold in the presence of TE_DEBS_ (Figure 3). Overall, this data demonstrates that PnG is capable of releasing products from an *in vitro* hexaketide system and even overcome limitations of the system that were caused by the chimeric TE_DEBS_ construct.

Surprised by this polyketide-releasing activity of PnG, we further asked whether PnG was not only able to release final products but also premature products (*i.e.*, di-, tri-, and tetraketides) from the Pn PKS pentaketide assembly line. Note that, in this system, the growing polyketide undergoes intramolecular translocation between PnA_V4_ and the first module of PnB and between the second module of PnB and PnC. In the case of inefficient module interaction and/or downstream translocation, the polyketide chain could become exposed to the ACP at the diketide (PnA_V4_-PnB transfer) and tetraketide (PnB-PnC transfer) level, where it might serve as a substrate for PnG. Indeed, PnG-dependent accumulation of di- and particularly tetraketide was observed, while no accumulation of triketide (transfer between the first and the second module within the PnB module) was detected (Figure S12). This supports that PnG is also able to release ACP-tethered polyketides during translocation in an *in vitro* setting.

## Final Discussion

Here, we characterized type II thioesterase PnG and studied the multiple effects of this enzyme on its cognate modular type I PKS assembly line *in vitro*. When incubated with different CoA- and ACP-bound acyl substrates, PnG showed high specificity for ACP-bound alkyl-residues compared to their (alkyl-)malonyl-ACP counterparts, with up to 1500-fold increased specificity constants (*k*_cat_/*K*_M_). Moreover, the enzyme did not hydrolyze CoA esters, indicating that PnG is specialized in removing aberrant ACP-tethered acyl residues from the phoslactomycin assembly line, thus increasing the productivity of phoslactomycin product formation.

Using a recently established *in vitro* Pn PKS system,^30^ we show that PnG indeed leads to a higher polyketide production in the presence of native but notably also non-native substrates. Addition of PnG in extender unit competition assays increases product formation by up to 14-fold and shapes the product profile toward the natural product. Further experiments suggest a 2-fold role of PnG. First, the enzyme clears the phoslactomycin assembly line that has become (partially) blocked through ACP misloading during protein expression. Maturation of the assembly line requires the transfer of free 4′PP onto the different ACPs of the Pn PKS. However, the corresponding 4′PP transferases are known to be promiscuous and also transfer different acylated-4′PP arms,^30,33−35^ which results in nonfunctional enzyme complexes, strongly limiting biosynthetic activity. Note that this clearing function of TEIIs is not only important *in vivo* but also for the *in vitro* reconstitution of fully active assembly lines, especially for subsequent kinetic studies. Beyond this initial clearing, PnG also acts on the active assembly line, where it preferentially removes nonproductive acyl-ACP extender units that accumulate during biosynthesis, either because of unspecific decarboxylation of ACP-tethered (alkyl)malonyl residues^36−38^ or because of slow processing of non-native extender units on the assembly line.

Notably, PnG does not only show hydrolysis activity toward short acyl-extender units but also toward ACP-bound polyketides. PnG releases intermediates and also final products, as demonstrated with the penta- and hexaketide system lacking a terminal thioesterase. This activity of TEIIs can be highly useful, especially in *in vitro* setups, where (chimeric) thioesterase fusions with the final domain are not active or might create artifacts, as demonstrated in the case of the hexaketide system.

Finally, while PnG generally shows beneficial effects on the *in vitro* system, it is important to note that the action of the enzyme is strongly concentration-dependent. This becomes especially apparent in the case of increased PnG concentrations. While low PnG concentrations improve the productivity of natural and non-natural polyketides in all cases, increasing PnG concentrations seem to discriminate stronger against the formation of non-natural products, most likely because of increased activity on non-native modifications bound to the ACPs. Thus, finely tuning the optimal concentration of TEIIs *in vitro* can be used to shape the product profile towards natural (*e.g*., through providing rather high TEII concentrations) and/or non-natural polyketides (*e.g*., through providing rather low TEII concentrations), which might be an interesting strategy for the production of native and modified polyketides in the future.

## Supporting Information Available

SDS-PAGE analysis of PnG and PnG S93G, kinetic measurements of PnG hydrolytic activity, phoslactomycin (Pn) PKS scheme, structures and masses of tetra- and pentaketides, effect of increasing PnG concentration on polyketide production, PnG catalytic knockout control PnG S93G, tandem mass spectrometric analysis of native and non-native pentaketides, impact of diketide SNAC thioester on PnG, CoA ester hydrolysis activity of PnG, effect of PnG on product profiles and pentaketide production, release of intermediates from Pn PKS pentaketide, and list of polyketide masses (*m*/*z*) (PDF)

## Accession Codes

PnA: AFJ05078.1 (NCBI Accession number); PnB: AFJ05079.1 (NCBI Accession number); PnC: AFJ05066.1 (NCBI Accession number); PnG: AFJ05065.1 (NCBI Accession number); DEBS thioesterase: 1MO2_ A (Uniprot ID); Npt: OSY40025 (Uniprot ID).
