# Supplementary material for: Multiple Functions of the Type II Thioesterase Associated with the Phoslactomycin Polyketide Synthase
Source: Biochemistry. 2022 Nov 15;61(23):2662–71. doi: 10.1021/acs.biochem.2c00234 (PMC9730843; doi:10.1021/acs.biochem.2c00234)
Supplement: Supplementary file 1 — bi2c00234_si_001.pdf [file bi2c00234_si_001.pdf]

Multiple functions of the type II thioesterase associated with the phoslactomycin polyketide synthase

Kyra Geyer [a], Steffen Hartmann [a], Randolph R. Singh [b], Tobias J. Erb [a], [c] *

[a]Department of Biochemistry and Synthetic Metabolism; Max Planck Institute for Terrestrial Microbiology, Karl-von-Frisch-Str. 10, D-35043 Marburg, Germany [b]Luxembourg Centre for Systems Biomedicine (LCSB), University of Luxembourg, avenue du Swing 6, L-4367 Belvaux, Luxembourg [c]LOEWE Center for Synthetic Microbiology (Synmikro), Karl-von-Frisch-Str. 16, D-35043 Marburg, Germany.

*corresponding author: toerb@mpi-marburg.mpg.de

Supplementary Information

Figures S1 – S12

Table S1

Phoslactomycin DNA sequences

References

Figure S1: SDS-Page analysis of PnG and PnG S93G. Insoluble fraction (1), lysate (2), Ni-NTA agarose elution (3) and the purified protein after size exclusion chromatography (4). The expected size of PnG is 30.2 kDa.

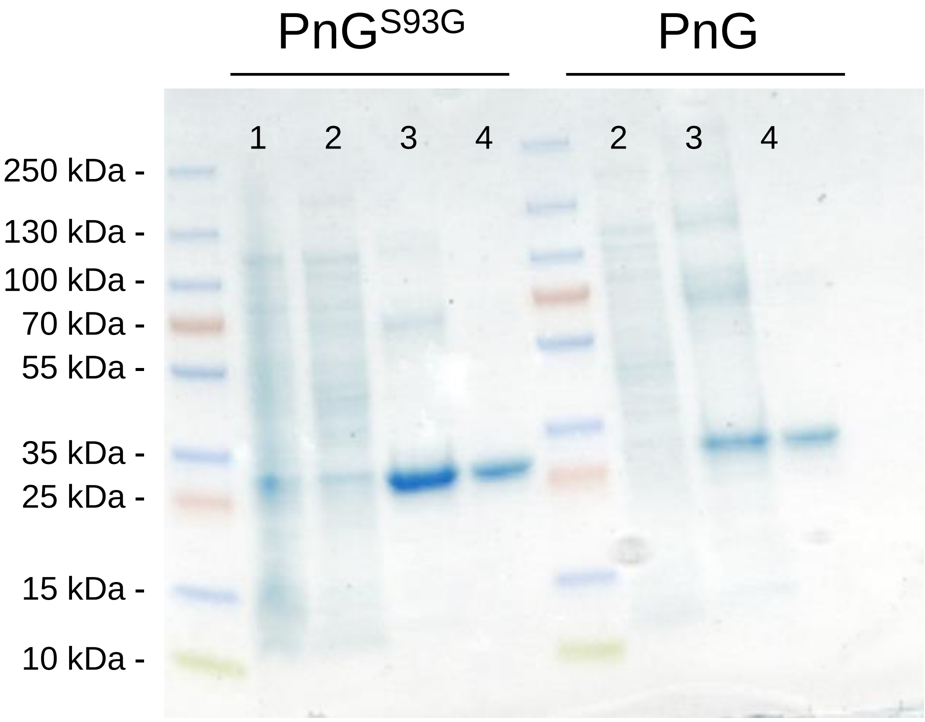

Figure S2: Kinetic measurements of PnG hydrolytic activity towards ACP bound acyl-residues fitted to Michaelis Menten plot (GraphPad Prism 8.0.0) **A)** malonyl-ACP_{LD}, **B)** acetyl-ACP_{LD}, **C)** ethylmalonyl-ACP_{LD} **D)** butyl-ACP_{LD}, **E)** malonyl-ACP2, **F)** acetyl-ACP2, **G)** methylmalonyl-ACP2, **H)** propionyl-ACP2, **I)** ethylmalonyl-ACP2, **J)** butyl-ACP2.

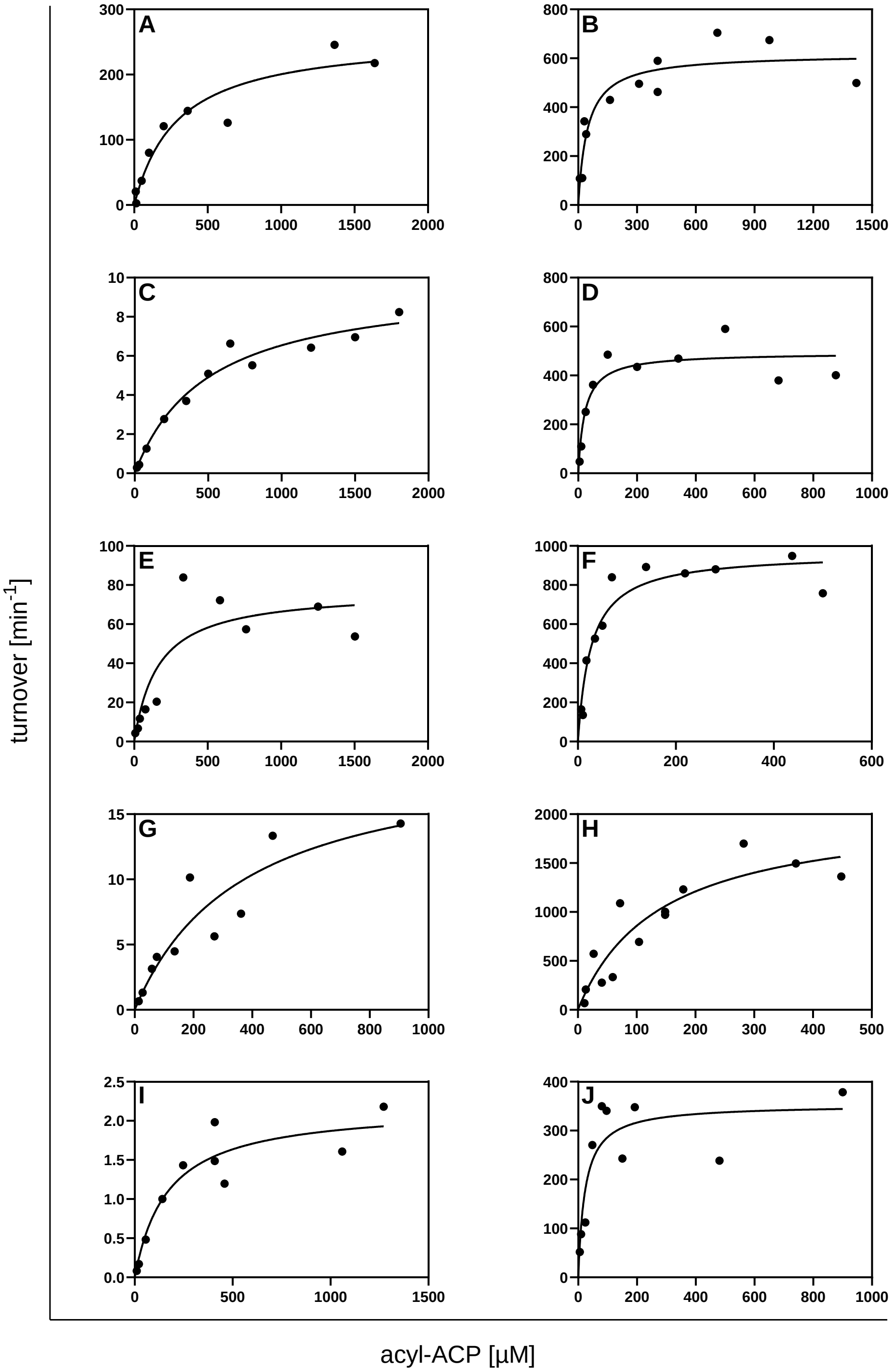

Figure S3: Phoslactomycin (Pn) PKS. ACP = acyl carrier protein; AT = acyltransferase; DH = dehydratase; KR = ketoreductase; TE = thioesterase; **1** = cyclohexanecarboxyl-CoA; **2** = malonyl-CoA; **3** = (2*S*)-ethylmalonyl-CoA; **7** = phoslactomycin polyketide backbone, **8** = bioactive phoslactomycin derivatives; **R** = isobutyloxy; isovaleryloxy; 4-methylcaleryloxy; cyclohexylcarbonyloxy; 4-methylheptanoyloxy. [1, 2].

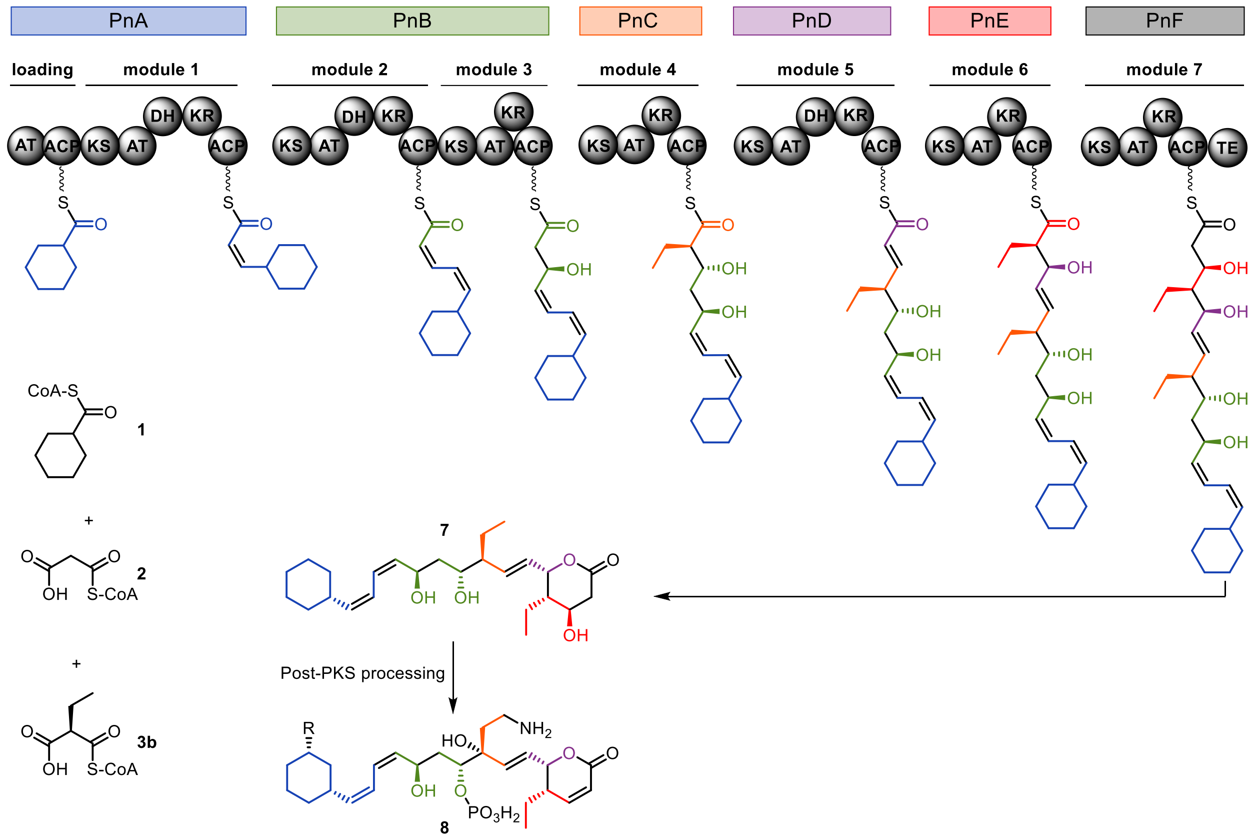

Figure S4: Structures and masses of tetra and pentaketides. (A) Depicts the tetraketide **4** released by the phoslactomycin system. **4** is dehydrated to **4.1**, the exclusive mass found for the tetraketide. **(B)** Depicts the pentaketide **5b** (assembled with ethylmalonyl-CoA as a substrate for PnC). **5b** is cyclized and subsequently oxidizes in solution.

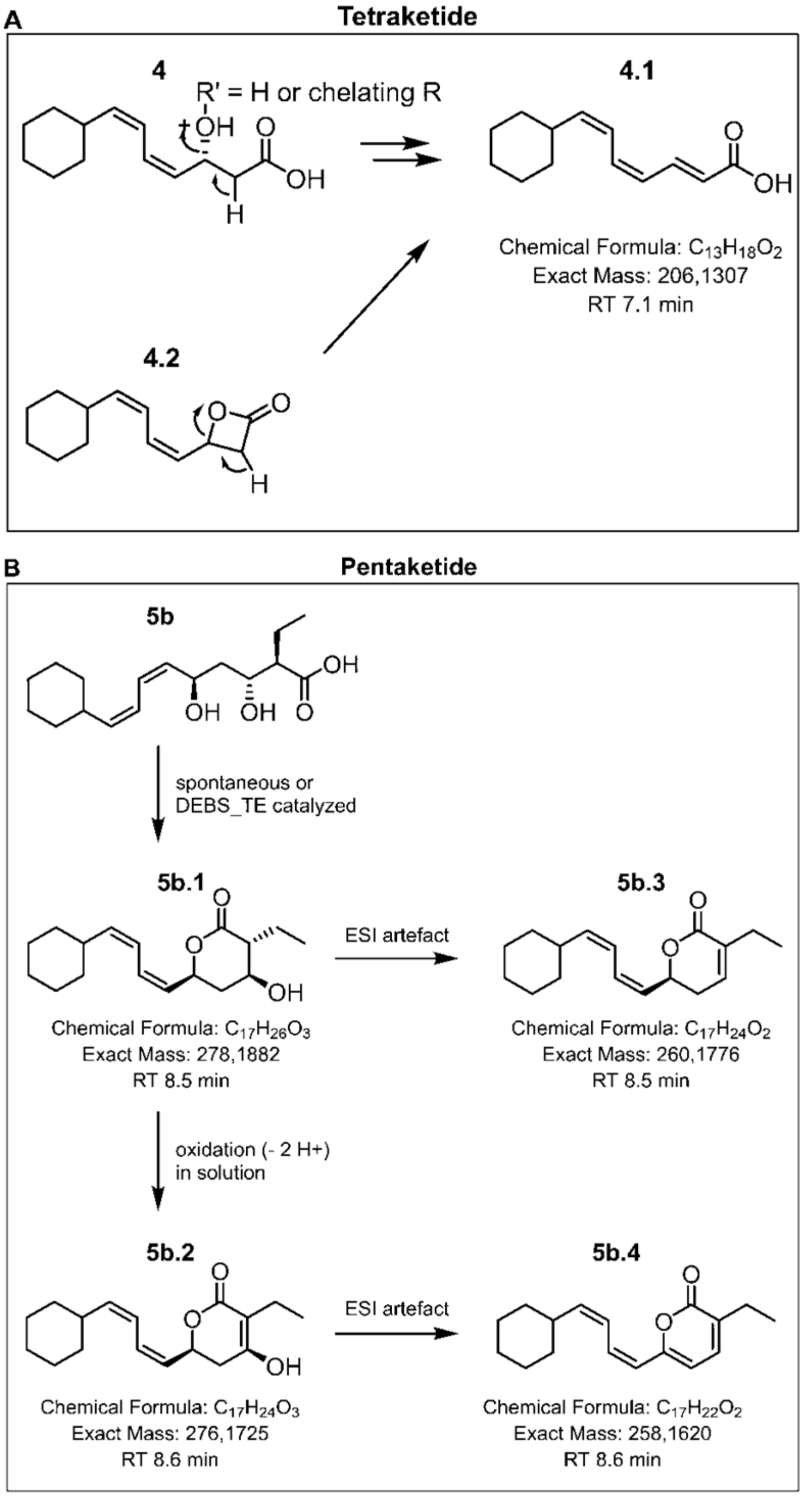

Figure S5: Effect of increasing PnG concentration on polyketide production after 2 h and overnight. All sample series were normalized to the control sample without PnG. Concentrations of PnG, indicated on the x-axis ranged between 0.1 and 2 molar equivalents of ACPs present in the assay. In **B**, **C** and **D** the heat map indicates the initial product distribution, set relative to the natural product **5b**. Residues indicated are: **a**=methyl-, **b**=ethyl-, **c**=butyl-, **d**=3-methlbutyl-, **e**=hexyl-residues. (**A**) Tetraketide production is increased up to five-fold. No effect of acetyl-CoA addition can be observed. (**B**) Pentaketide production in reaction mixtures containing malonyl-CoA and one *α*-substituted malonyl-CoA derivative as extender units. Increase in product formation can be seen for all pentaketide derivatives **5a-e**. The strong increase of **5a** can be explained by the very little initial amounts close to the detection limit. The overall lower fold-changes in the overnight samples could be explained by the consecutive biosynthesis of pentaketides in the samples not containing PnG, while samples containing PnG ran out of resources. (**C**) Pentaketide production run under competitive reaction conditions, containing malonyl-CoA and all five *α*-substituted malonyl-CoA extender units. Initial product amount of **5b** is highest, as well as the fold-change when PnG is added. (**D**) Pentaketide production with pre-treated Pn PKS enzymes. Treatment with PnG ensures removal of all potentially aberrant acyl-residues. PnG is removed by size exclusion chromatography from the Pn PKS enzymes. Data points are from multiple biological (**A**, **B** o.n, **C**, **D**) and technical (**A**, **B**, **C**, **D**) replicates.

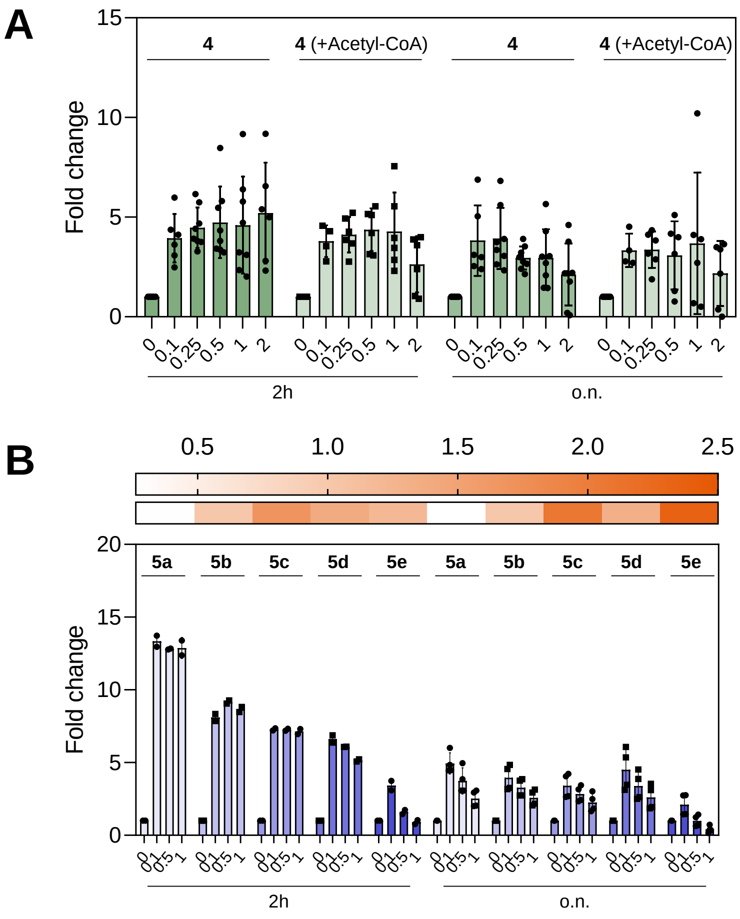

C

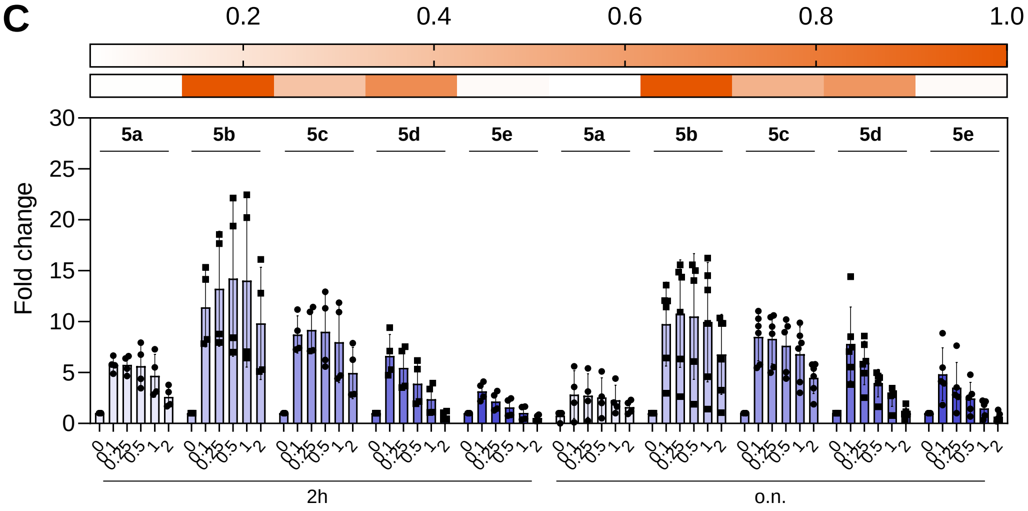
0.2
0.4
0.6
0.8
1.0
Fold change
30
25
20
15
10
5
0
5a
5b
5c
5d
5e
5a
5b
5c
5d
5e
0
0.1
0.25
0.5
1
2
2h
o.n.

D

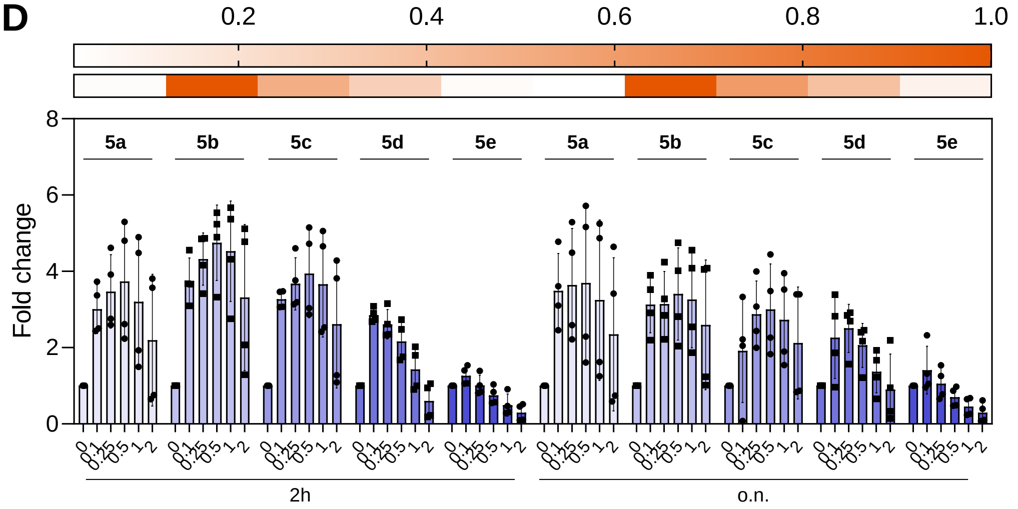
0.2
0.4
0.6
0.8
1.0
Fold change
8
6
4
2
0
5a
5b
5c
5d
5e
5a
5b
5c
5d
5e
0
0.1
0.25
0.5
1
2
2h
o.n.

Figure S6: PnG catalytic knockout control PnG S93G. The active site serine is mutated to a glycine, rendering it unable to covalently bind the substrate. This control was performed to assure that all effects, observed in the assays are due to PnG and not caused by protein contaminations. Shown here is the relative product amount of competitive pentaketide assays containing PnG S93G compared with assays containing no PnG. Samples containing PnG S93G, taken after 2 h and o.n., contain the same product amounts as samples without the type II thioesterase PnG. Residues indicated are: **a**=methyl-, **b**=ethyl-, **c**=butyl-, **d**=3-methlbutyl-, **e**=hexyl-residues.

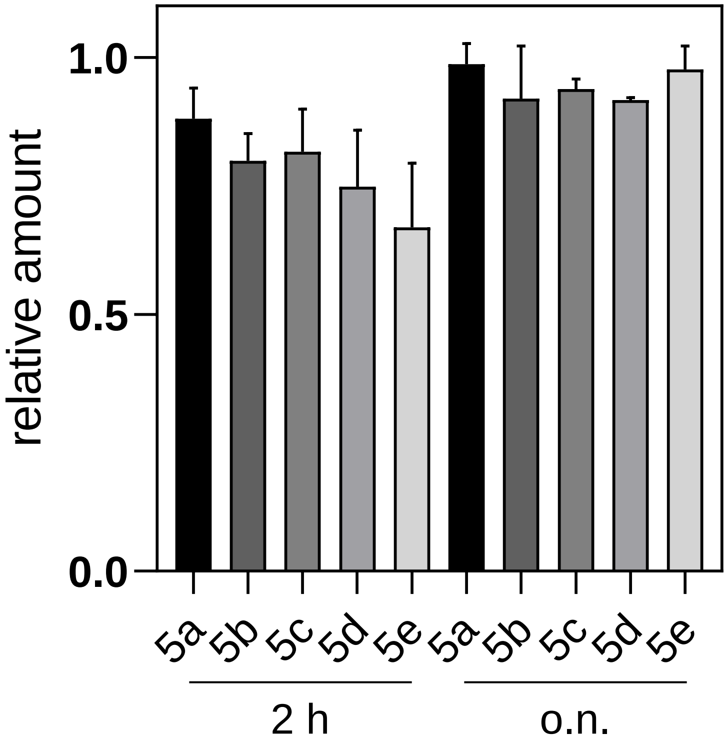

Figure S7: Tandem mass spectrometric analysis of native and non-native pentaketides. Putative structures for the different polyketides were generated using knowledge on the biosynthetic logic of the phoslactomycin PKS system. The fragment masses were obtained by inspecting the data files using Xcalibur Qual Browser software. The experimental fragments were used to verify the proposed structure of the molecules by drawing fragment structures to explain the observed fragment mass. The structures of the fragments were drawn using ChemDoodle software. All precursor ion and fragment ion masses differed by less than 5 ppm compared to the theoretical mass. In addition, *in-silico* fragmentation was performed using MetFrag online. However, the top scoring candidates had molecular formulas that are unnatural (eg. Contains Boron or Fluorine, or Halogen) and are not expected to be formed by the Pn PKS *in vitro*. **A)** Fragmentation pattern of **5a** (methylmalonyl-CoA incorporation), **B)** Fragmentation pattern of **5b** (native product, ethylmalonyl-CoA incorporation), **C)** Fragmentation pattern of **5c** (butylmalonyl-CoA incorporation), **D)** Fragmentation pattern of **5d** (3-methylbutylmalonyl-CoA incorporation) **E)** Fragmentation pattern of **5e** (hexylmalonyl-CoA incorporation)

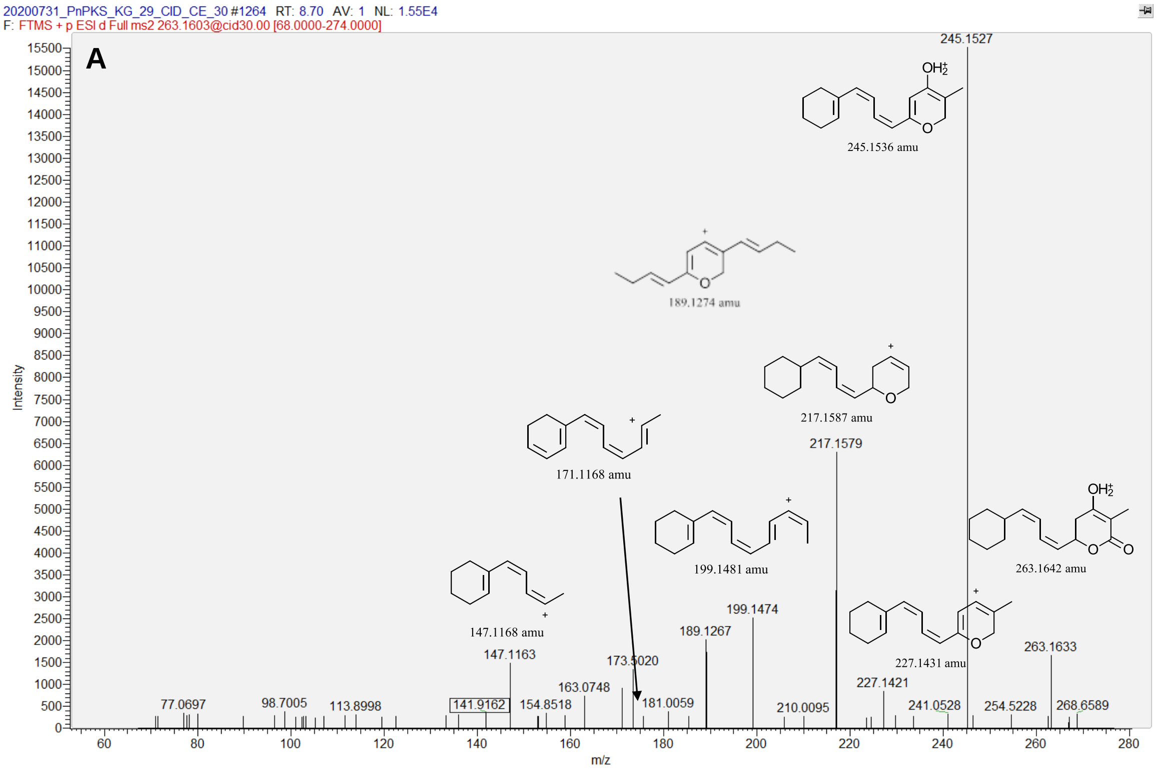

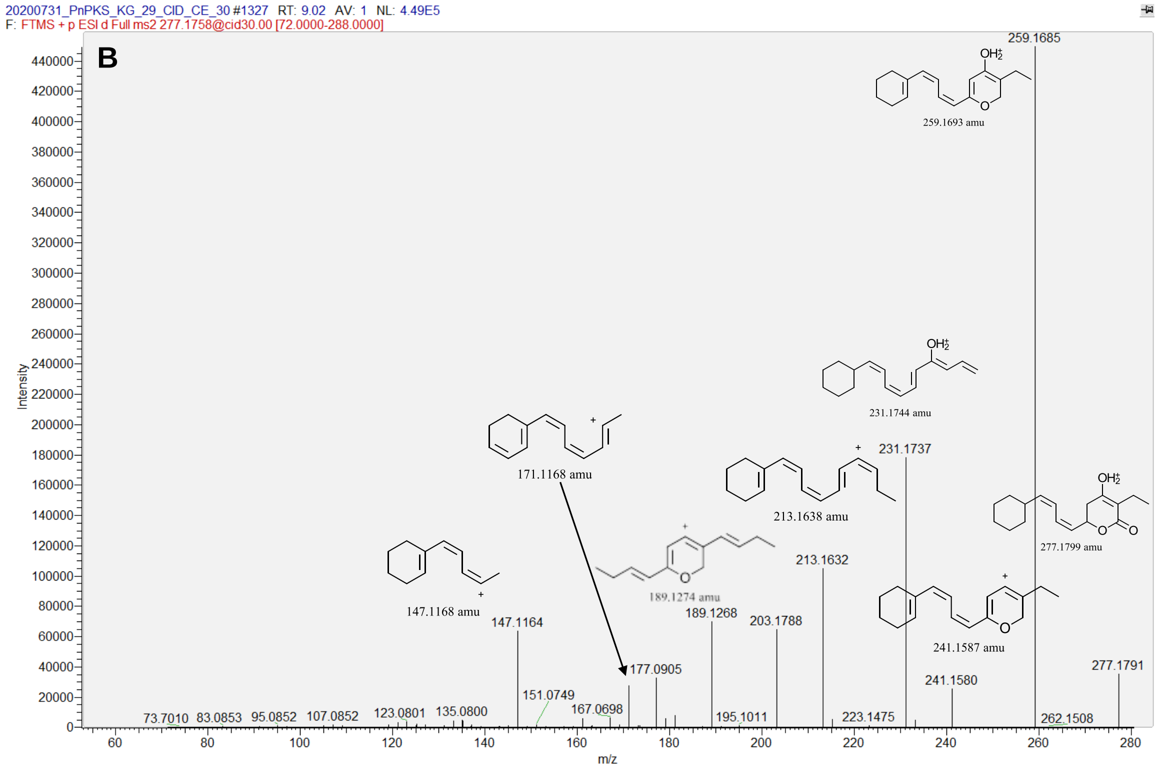
20200731_PnPKS_KG_29_CID_CE_30 #1327 RT: 9.02 AV: 1 NL: 4.49E5
F: FTMS + p ESI d Full ms2 277.1758@cid30.00 [72.0000-288.0000]
B
259.1685
259.1693 amu
231.1744 amu
171.1168 amu
231.1737
213.1638 amu
277.1799 amu
147.1168 amu
189.1274 amu
213.1632
147.1164
189.1268
203.1788
241.1587 amu
177.0905
277.1791
241.1580
151.0749
167.0698
73.7010
83.0853
95.0852
107.0852
123.0801
135.0800
195.1011
223.1475
262.1508
Intensity
m/z

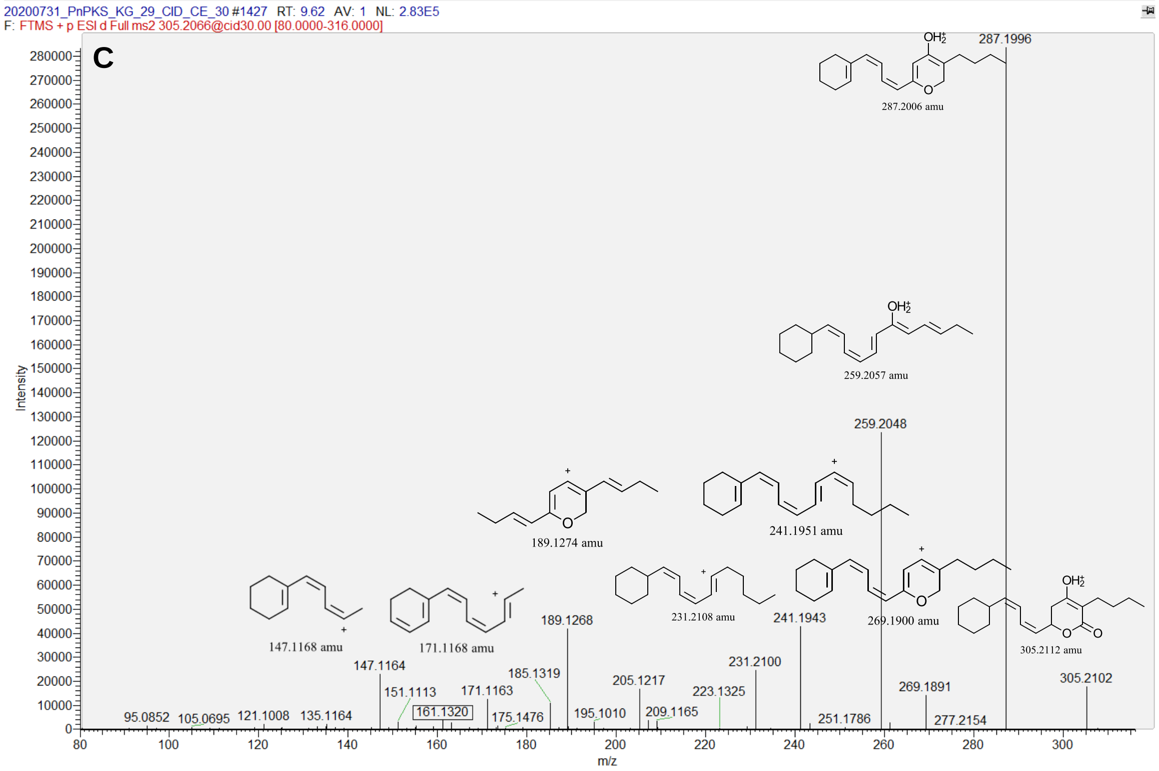
20200731_PnPKS_KG_29_CID_CE_30 #1427 RT: 9.62 AV: 1 NL: 2.83E5
F: FTMS + p ESI d Full ms2 305.2066@cid30.00 [80.0000-316.0000]
C
287.1996
287.2006 amu
259.2057 amu
259.2048
189.1274 amu
241.1951 amu
231.2108 amu
241.1943
269.1900 amu
305.2112 amu
147.1168 amu
171.1168 amu
189.1268
147.1164
185.1319
231.2100
205.1217
223.1325
269.1891
305.2102
151.1113
171.1163
161.1320
175.1476
195.1010
209.1165
251.1786
277.2154
95.0852
105.0695
121.1008
135.1164
Intensity
m/z

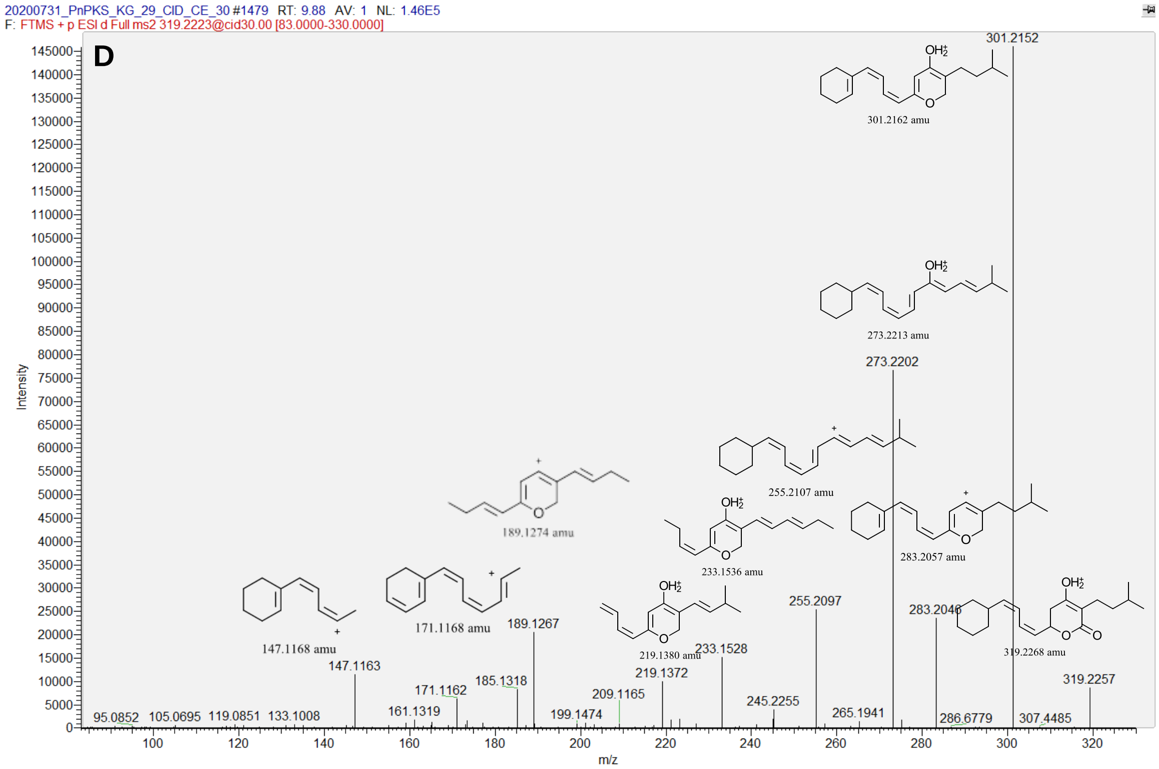
20200731_PnPKS_KG_29_CID_CE_30 #1479 RT: 9.88 AV: 1 NL: 1.46E5
F: FTMS + p ESI d Full ms2 319.2223@cid30.00 [83.0000-330.0000]
D
Intensity
m/z
301.2152
301.2162 amu
273.2213 amu
273.2202
255.2107 amu
189.1274 amu
233.1536 amu
283.2057 amu
171.1168 amu
189.1267
255.2097
283.2046
147.1168 amu
219.1380 amu
233.1528
319.2268 amu
147.1163
219.1372
319.2257
185.1318
209.1165
171.1162
245.2255
161.1319
199.1474
265.1941
95.0852
105.0695
119.0851
133.1008
286.6779
307.4485

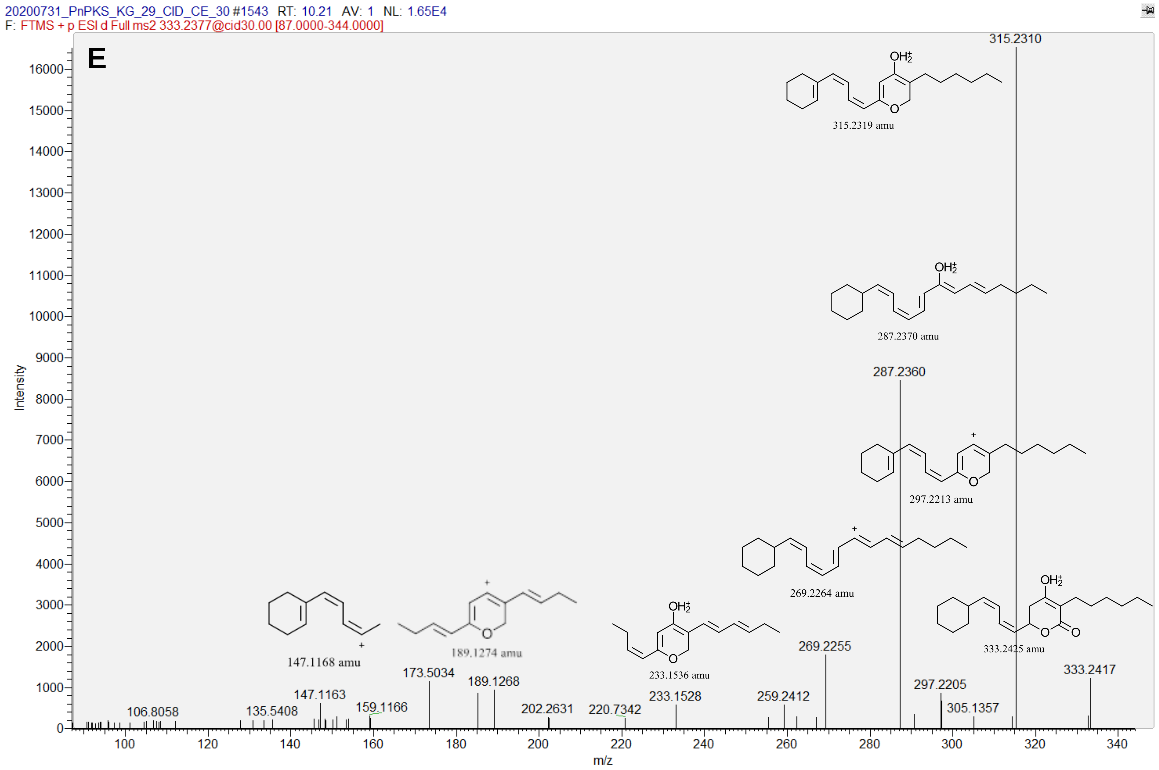
20200731_PnPKS_KG_29_CID_CE_30 #1543 RT: 10.21 AV: 1 NL: 1.65E4
F: FTMS + p ESI d Full ms2 333.2377@cid30.00 [87.0000-344.0000]
E
Intensity
m/z
315.2310
315.2319 amu
287.2370 amu
287.2360
297.2213 amu
269.2264 amu
333.2425 amu
147.1168 amu
189.1274 amu
233.1536 amu
269.2255
173.5034
189.1268
333.2417
147.1163
297.2205
233.1528
259.2412
106.8058
135.5408
159.1166
202.2631
220.7342
305.1357

Figure S8: **Diketide SNAC thioester impacts PnGs activity**. The x-axis in **A** and **B** indicates the concentration of PnG, equimolar towards the ACPs present, samples taken after 2 h. (**A**) Pn polyketide production can be initiated by PnA_{V4} (panel 1). Alternatively, PnA_{V4} can be bypassed when the respective diketide SNAC analog, serving as a substrate for PnB is added (panel 2). When initiated with PnA_{V4} tetraketide production increased five-fold, surprisingly when bypassing PnA_{V4} and supplying the SNAC ester, no effect of PnG was detected. When producing the pentaketide **5b**, a 14-fold increase in production upon PnG addition is observed, however when bypassing PnA_{V4} **5b** production increases maximal seven-fold. Either PnG has a differential effect on PnA_{V4}, PnB and PnC-TE_{DEBS} or the SNAC-ester has an inhibition-like effect on PnG. (**B**) To test if the diketide-SNAC analog is responsible for the decrease of PnGs effect on polyketide production, we ran **5b** production assays with PnA_{V4}, PnB, PnC-TE_{DEBS} (a), PnB, PnC-TE_{DEBS} (b) and PnA_{V4}, PnB, PnC-TE_{DEBS} plus the diketide-SNAC ester (c). A six-fold increase was observed in a, however in b and c an increase of only approximately four-fold was measured. This leads to the assumption that the diketide SNAC analog impacts PnG activity. SNAC thioesters also serve as substrates for thioesterases, however with a slower turnover. PnG most likely binds the SNAC ester as substrate leading to a less strong effect on Pn PKS.

A

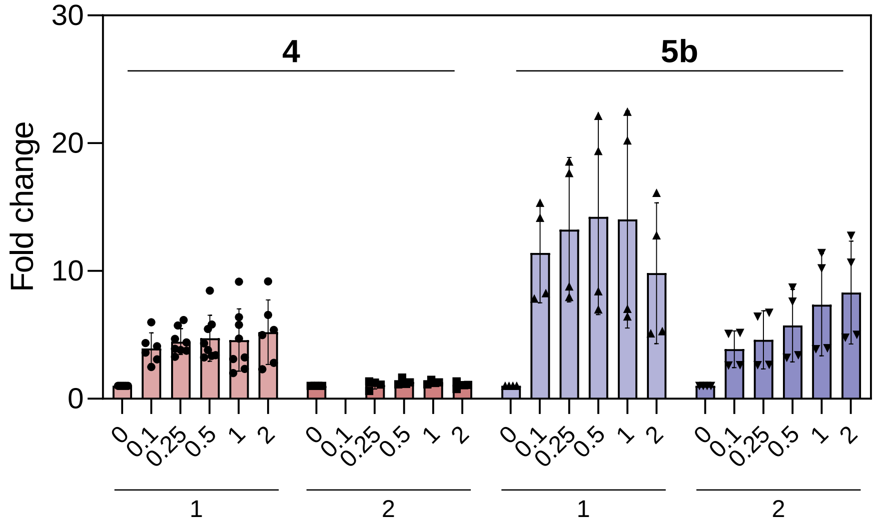

B

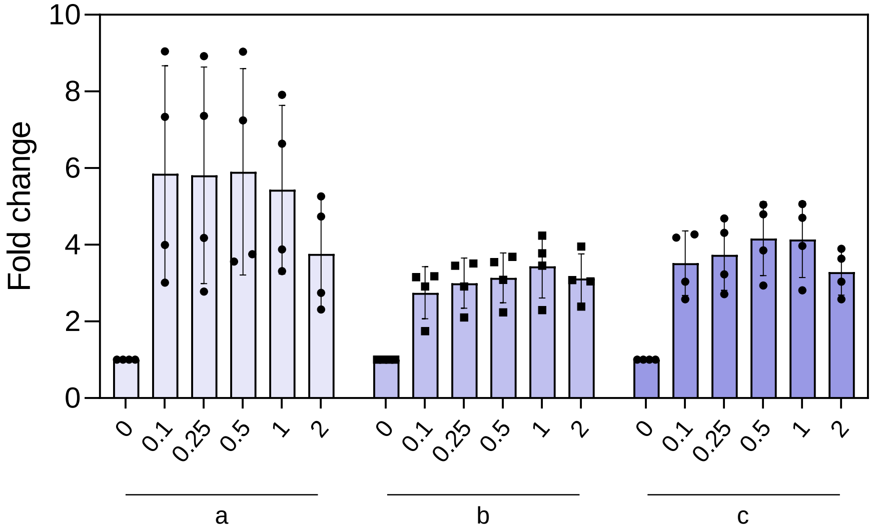

Figure S9: PnG does not hydrolyze CoA-thioesters. Incubation of PnG with the CoA-thioester substrates present in the polyketide production assays. Samples from assays containing PnG and without PnG as negative control were taken after 0 h, 2 h and 4 h. Neither buildup of free CoA-SH, nor depletion of the acyl-CoA thioester can be observed. **A)** Cyclohexanecarboxyl-CoA with PnG, **B)** and without PnG. **C)** Malonyl-CoA with PnG, **D)** and without PnG. **E)** Methylmalonyl-CoA with PnG, **F)** and without PnG. **G)** Ethylmalonyl-CoA with PnG, **H)** and without PnG. **I)** Butylmalonyl-CoA with PnG, **J)** and without PnG. **K)** 3-methyl-butylmalonyl-CoA with PnG, **L)** and without PnG. **M)** Hexylmalonyl-CoA with PnG, **N)** and without PnG.

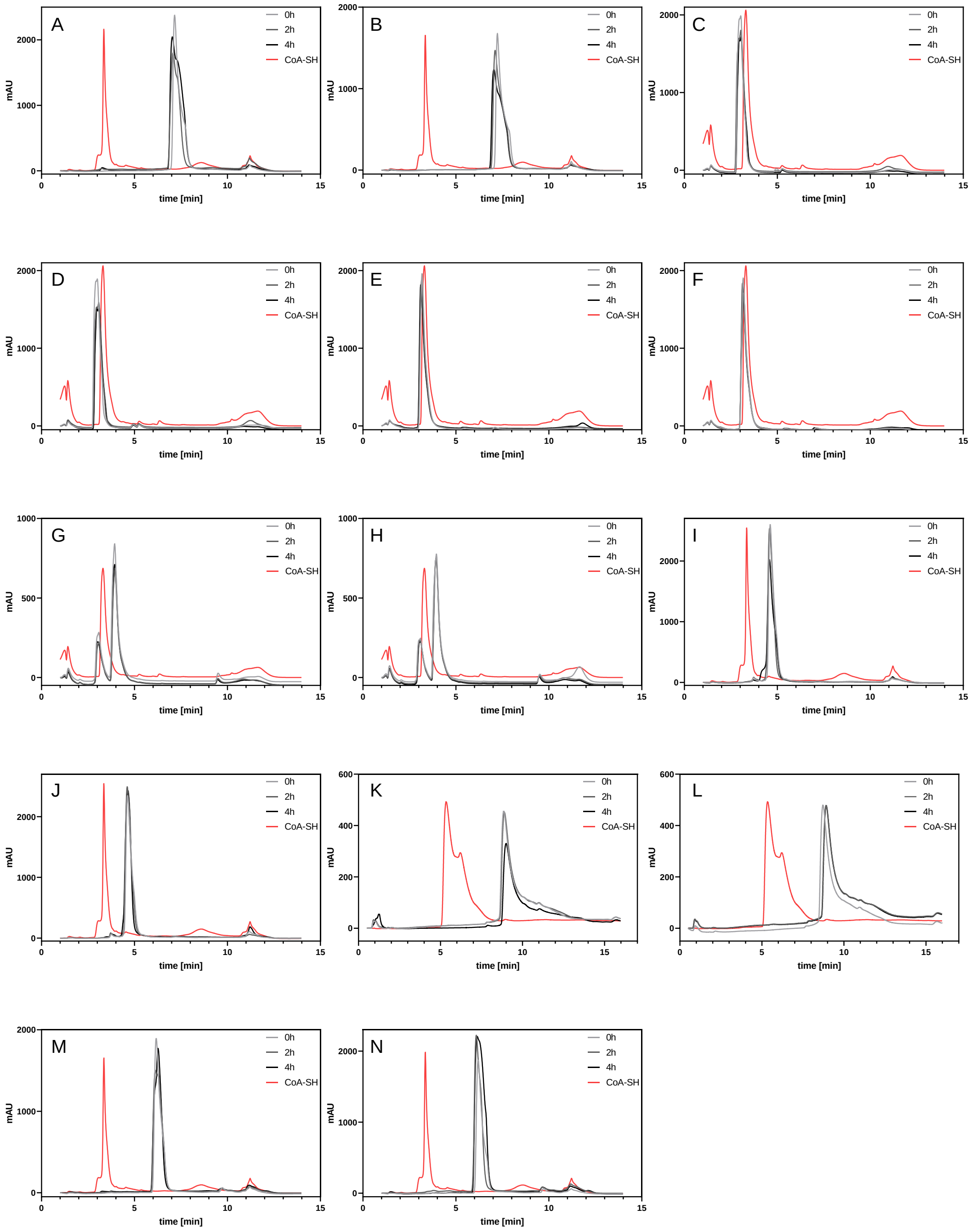

Figure S10: Effect of PnG on the product profile in competitive pentaketide assays. All samples are normalized to the **5b** ion count in the same sample. When comparing the products in competitive assays it shows that all alternative products relatively decrease upon addition of PnG (while the absolute amount of product increases for all – compare to Figure S4). This shaping in product profile towards the natural product **5b** could be caused a longer exposure of non-native acyl-residues due to slower processing. The longer exposure results in a higher probability of PnG to hydrolyze the residue, which increases with higher PnG concentration. Shown are the values for two biological replicate with each two technical replicates.

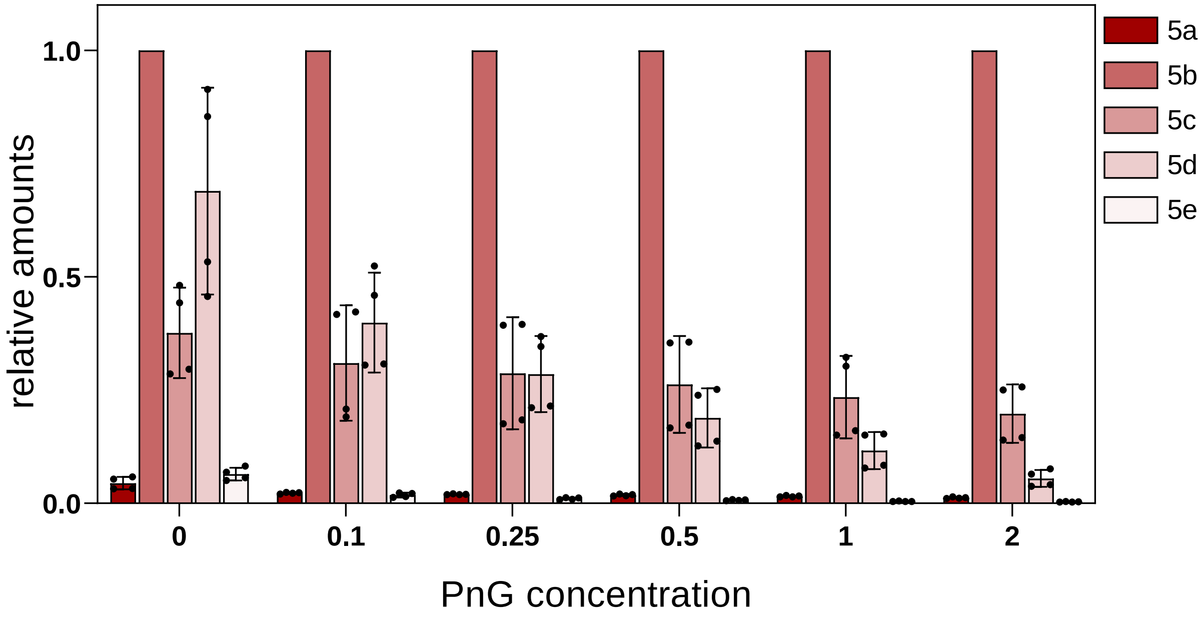

Figure S11: Effect of PnG on the pentaketide production and release without terminal thioesterase. Pn PKS assembly line is missing the terminal thioesterase (PnA_{V4}-PnB-PnC). Residues indicated are: **a**=methyl-, **b**=ethyl-, **c**=butyl-, **d**=3-methlbutyl-, **e**=hexyl-residues. Concentrations of PnG ranged between 0.1 and 2 molar equivalents of ACPs present in the assay. Pentaketide production was run under competitive reaction conditions, all mixtures contained malonyl-CoA and all *α*-substituted extender units. **A)** Pentaketide production after 2 h and overnight (o.n.) incubation of the assay. All sample series were normalized to the control sample without PnG. The heat map displays the product distribution of non-native pentaketides (**5a**, **5c-d**) compared to the native pentaketide **5b** in absence of PnG. A strong increase of product formation after 2 h can be observed for all pentaketides, particularly **5b-5c** (200- to 100-fold increase respectively). The effect of PnG on product formation becomes less prominent in the overnight samples. This is most likely due to slow but consistent production of pentaketides in the assays not containing PnG. **B)** Heat map of production comparison of assays terminating without (PnA_{V4} + PnB + PnC) and with a terminal thioesterase (PnA_{V4} + PnB + PnC-TE_{DEBS}) to identify if PnG can functionally reconstitute the terminal TE, samples after 2 h and overnight (o.n.). In absence of PnG product formation reaches only 5% of the level when a terminal TE is present. This supports the importance of the fusion of TE_{DEBS} to PnC for pentaketide release. Upon addition of PnG final product amount in ion counts reaches up to approx. 90% (**5b**), 80% (**5c**) and 55% (**5d**) of that, if TE_{DEBS} was present. PnG is able to hydrolyze the pentaketide product bound to PnC.

A

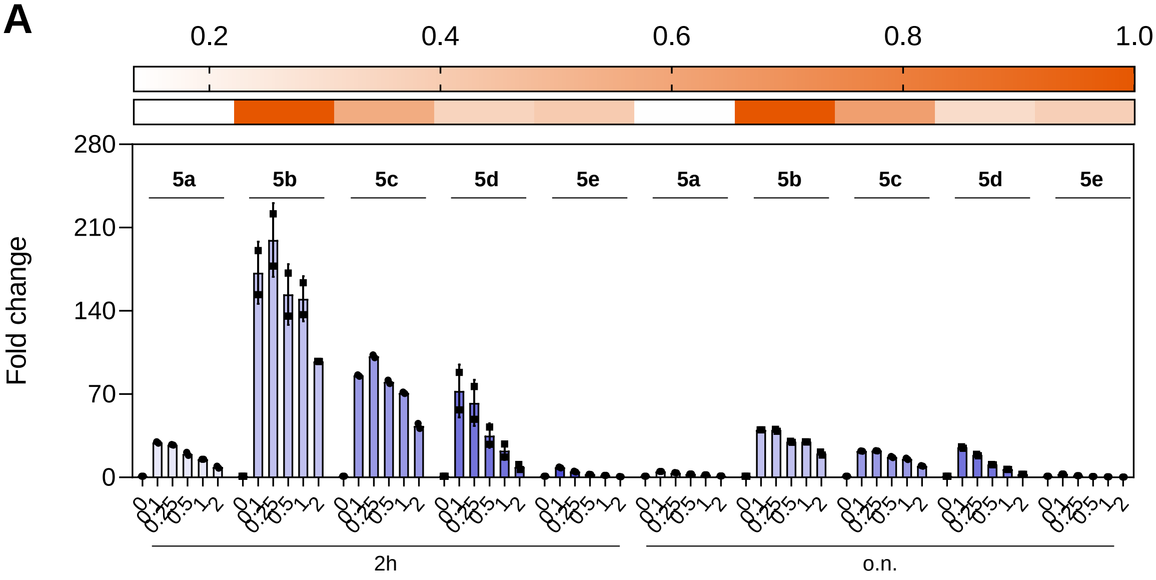

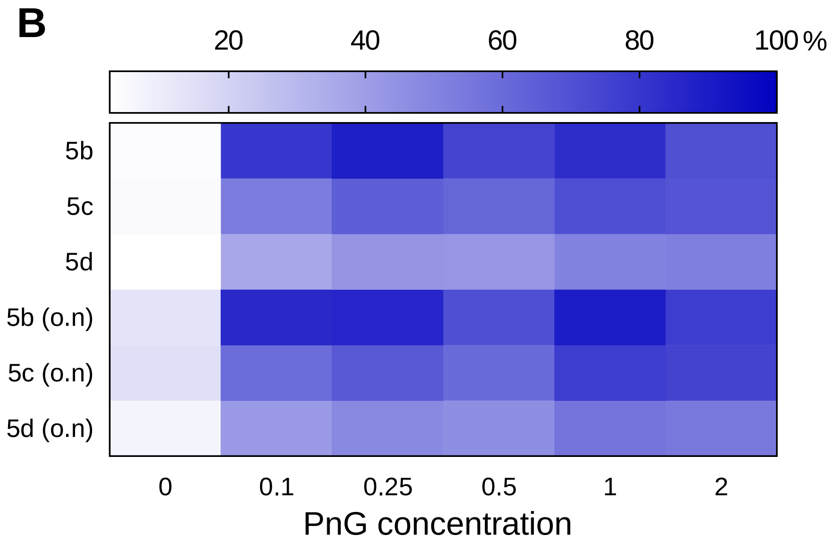
B
20
40
60
80
100 %
5b
5c
5d
5b (o.n)
5c (o.n)
5d (o.n)
0
0.1
0.25
0.5
1
2
PnG concentration

Figure S12: Release of intermediates from Pn PKS pentaketide system. Initiation with PnA_{V4} and termination with $PnC\text{-}TE_{DEBS}$. Shown are the peak areas of samples with increasing PnG concentration relative to the control without PnG. Concentrations of PnG ranged between 0.1 and 2 molar equivalents of ACPs present in the assay. CHC=cyclohexanecarboxylic acid ($C_7H_{12}O_2$; calculated: *m/z* [H^+]: 129.091; *m/z* [Na^+]: 151.073; found: *m/z* [Na^+]: 151.0758; retention time 8.5 min), diketide ($C_9H_{14}O_2$; calculated: *m/z* [H^+]: 155.1067; *m/z* [Na^+]: 177.0886; found: *m/z* [Na^+]: 177.0926; retention time 8.5 min), triketide ($C_{11}H_{16}O_2$; calculated: *m/z* [H^+]: 181.1223; *m/z* [Na^+]: 203.1043; found: *m/z* [Na^+]: 203.1088; retention time 9 min), tetraketide ($C_{13}H_{20}O_3$; calculated: *m/z* [H^+]: 225.1485 ; *m/z* [Na^+]: 247.1305; dehydrated $C_{13}H_{18}O_2$; *m/z* [H^+]: 207.138; *m/z* [Na^+]: 229.1199; found *m/z* [H^+] 207.1397; retention time 7 min). No difference can be observed between the 2 h and overnight (o.n.) samples. Release of triketide cannot be observed. The tri- and tetraketide are produced by the bi-modular protein PnB. The triketide is internally translocated to the downstream module, protecting it from PnG.

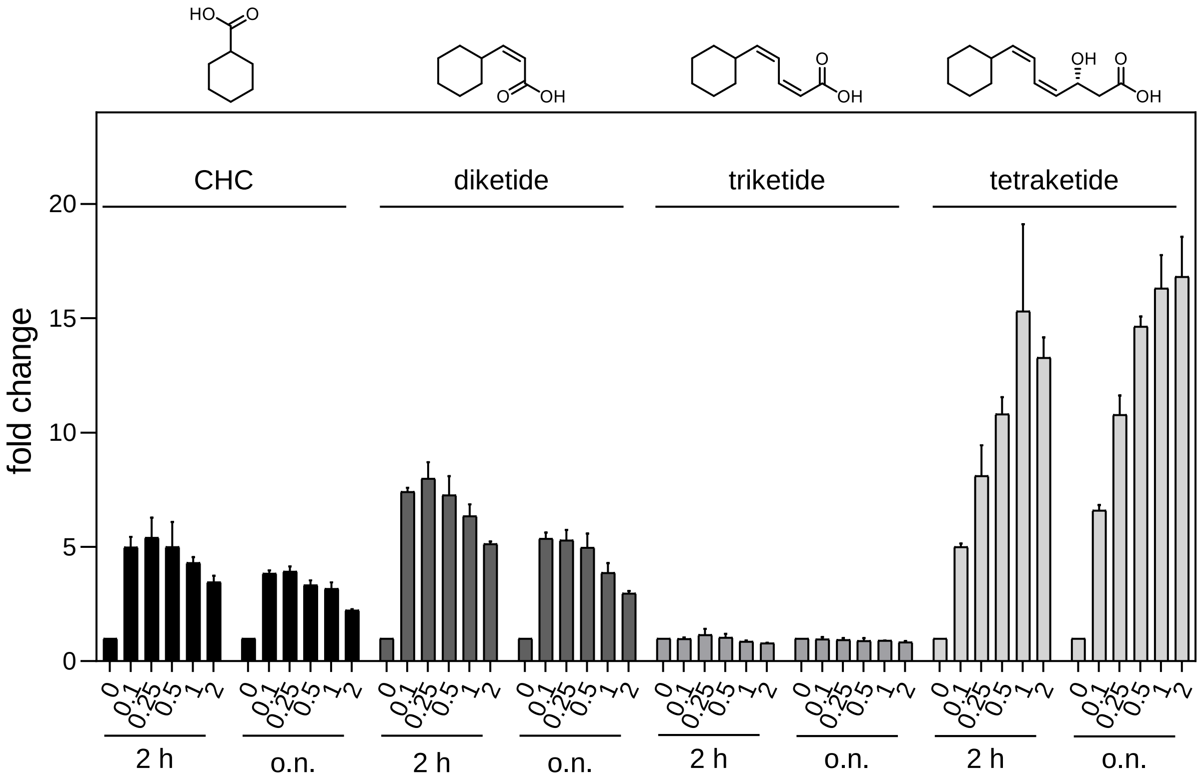

Table S1: List of polyketide masses (*m/z*) from the *in vitro* production assays. Both, *m/z* calculated and detected are given together with the retention time. **4** corresponds to the tetraketide, **5** to the pentaketide. **a** indicates methyl-, **b** ethyl-, **c** butyl-, **d** 3-methyl-butyl and **e** hexyl-residue.

**Compound**	**Retention time [min]**		**[H⁺] *m/z* calculated**	**[H⁺] *m/z* detectedd**	**Δ *m/z***	**[Na⁺] *m/z* calculated**	**[Na⁺] *m/z* detectedd**	**Δ *m/z***
**4**	7.1		207.1380	207.1403	0.0023	229.1199	-	
**5a**	8.1		263.1642	263.1648	0.0006	285.1461	285.1316	0.0145
	8.1		245.1536	245.1546	0.0010	267.1355	-	
**5b**	8.5		277.1798	277.1818	0.0020	299.1618	299.1635	0.0017
	8.5		259.1693	259.1710	0.0017	281.1512	-	
**5c**	9.2		305.2111	305.2130	0.0019	327.1931	327.1948	0.0017
	9.2		287.2006	287.2025	0.0019	309.1825	-	
**5d**	9.5		319.2268	319.2287	0.0019	341.2087	341.2105	0.0018
	9.5		301.2162	301.2180	0.0018	323.1982	-	
**5e**	9.8		333.2424	333.2439	0.0015	355.2244	355.2313	0.0069
	9.8		315.2319	315.2330	0.0011	337.2138	-	

Phoslactomycin DNA sequences

DNA sequences of the Pn modules used for this study, as published in [4]. Individual domains within the different modules are highlighted in colors. Highlighted in red and italics are the linker sequences that are replaced with DEBS_TE (see below) to create PnB-TE$_{DEBS}$ PnC-TE$_{DEBS}$ and/or PnD-TE$_{DEBS}$.

PnAV4 (PnA Accession number AFJ05078

ATGGTAGCTGTAGCACTCGCACTGGAAACCGCTGATGCGGAACATTACGGCCCACTGTCCGACGACGCTGAAGAACGCGTTGCAGAGCTGCGTGCACTGCGCCCTCGTGA
CCGCACCTCCGCTCCGGATGCTACGTCTACCTGCGCAGTACCGCTGCTGCTGTCTGGTCGCACTGACGCAGGCCTGCGTGCTCACGCAGGTCGTCTGGCAGGTCATCTGC
AAGAACGTACAGACATCTCCCTGTCTGATGCAGCATTCACCCAGGCTACTACTCGCACTCTGTGGCCGCACCGTGCAATGGTGACCGCAGCCGACCGCGACGCTGTTGTTG
CTGCGCTGCGCGCTGTTGCCGAAGGTCGTGCTTCTGACGCAGCTGTACGCGCTGGTAGTGGTGGTCCGGAATCCGCTGTTTTCGTTTTCCCGGGCCAGGGTGCGCAGTGG
GTAGGCATGGCACGTGAACTGGCTGCTGCCGCTCCGGTATTCCGTGACAAACTGGCTGAGTGTGCTCGTGAACTGCGCCCGTTCGTTGACTTCGAACTGGACGACGTTCTG
TCTGGTGCCCTGCCGCTGGAACGTGTTGAAGTTATCCAGCCGGCTCTGTTCGCCGTAATGGTTAGCCTGGCGGAACTGTGGCGTTCCAACGGCGTTACGCCGGCTGCTGT
TGTGGGTCACTCTTTCGGTGAAATCGCTGCGGTTACTGCGGCAGGCGCTCTGACACTCGCAGACGGCGCGCGTCTGGTTGCTGCTGTATCCAAAGCTCTGGCACAACTGC
AAGGTAACGGCGAGATGGTTGCAGTAGCGCTGCCGGATGCTCAGGTAACCGAACTGGTTGCAGAATGGGACCTGGACCTGGACATCGCAGTTGTAAACGGCCCGCGTTCT
ACCGTGGTAGCTGGCCCAACCGAAGCAGCTACCGCTCTGGTTGAACGCCTGCGCGACCGCGATGTACGTGCAACTCTGCTGCCTATCGGTATCGCTGGTCACAGCCGCCG
TATGGAACCGGCACACGCATACCTGGTACAGGAGGCGTCTGCTGTCCGTCCGCGTGCTACTGGTATCCCAGTATACACATCTACCACTACCGACCCGCTGGACACTGGTGA
CCTCGACGCCGAACACTGGTTTCATTCTCTGCGCGAACCAGCACGTTTCCAGCAGGTTATCGAAGAGCTGCTGGGTCAGGGTCACCGCGTATTCGTAGAAATGTCCCCGCA
TCCGGTTCTGGCTCTGTCTATCGAAGAAACCGCAGCTCACCTGGGTCGTGATGTAGTAGTTCTGGAAACGATGCGTCGTGACGATGCGGGTCACGACCGTTATCTGCGTGC
TCTGGCGGAAGCACATCTGCACGGTGTTGCGCCGGACTGGTCTACTGTTCTGCCGGATGCTCGTCGTGTGACTCTGCCGCCGTACCGTCTGGACCTGGACACAGCTGACG
CTGCTGGCTCTGGCGGCACCGAACCGGGTGCCGGTCTCCGTGAACGCCTGCTGGGCGTTGCACCTGAACGTCGCATCGAAGAAGCCGTGCGTCTGGTTGTTGACGCTGT
GGCAGCACACATCGAACCGTCTGCTACAGCTATTGGTGCAGACCAGGCATTCCGTAGCTTAGGCGTTGACTCCGCTGGCGCACTGCGTGTGCGCAACCGTCTGGTAGAAA
CTACCGGCCTGCGCCTGCCGGCGACTATCCTGTTCGACCACCCGACCCCGCGTGCTCTGGCCGAAGAGCTGGTTCGCGCTGTACTGGGCGGTGACGAACCGGCACCGGC
AGCTACCGCTCCGGCTGACCCGGACGAGCCGCTGGCAATCGTAGGTATGGCGTGCCGTTTTCCAGGTGGTGTGGGTAGCCCGGACGAACTGTGGCAGCTCGTACACGAA
GGCGGGGATGCCGTTGCGGCCTTCCCGGCAGATCGTGGATGGGACCTGACCGCTCTGTACGACTCTGATGCCTCCCAGCCGGGCACCTCTTACCAGCGTGAGGCTGCAT
TACTGGACGGTGTAGCTGAGTTCGACGCTGCATTCTTCGGTATCTCTTCTCGTGAGGCTCTCGCAATGGACCCACAGCAGCGTCTGCTGCTCGAAACTTCTTGGGAGGCTC
TGGAACGTGGCGGTATTGACAGCACTACCCTGCGTGGTTCCCGCACTGGCGTTTTCACTGGTGTTATGTCTCTGCCGTACGGCCGCCCGCTCCATCAGGCGCGTCCGGAC
CTGGAGGGTTACGTAATGACTGGTACAACTTCTTCCGTGGTTTCTGGCCGTCTGTCTTATGTTTTAGGTCTGGAAGGTCCGGCTCTGACTCTGGACACCGCTTGCTCTAGTT
CTCTGGTTGCTCTGCACTTGGCAGGTCAGTCCCTGCGTCGTGGTGAATCTGACCTGGCTCTGGTCGGCGGTGCAACTGTGATGGCTGAACCGGGCCTGTTCATCGAGTTCT
CTCGTCTGCGCGCTCTGGCTCCGGACGGCCGTAGCAAGCCATTCAGCGCGGACGCTGACGGTTTCGGCATGGCAGAGGGTGTAGCTGTTCTGGTTGTTGAACGCCTGTCT
GACGCACGCCGTCTGGGTCACGACGTACTGGCCGTAGTTCGCGGTTCCGCTGTGAACCAGGACGGCGCTTCTAACGGTCTGACCGCACCGAACGGCCCGGCTCAGCAGC
GTGTAATTCGCGCTGCTCTGGACTCCGCAGGTCTGGTGCGGCTGACATCGATGTGGTTGAAGCACACGGCACAGGCACCCGCTTAGGCGACCCGATCGAAGCTCAGGCTC
TGATCGCTACTTACGGTAGCGACCGTCCGGCAGAACGTCCGCTGTACGTTGGTTCTCTCAAATCTAACATCGGCCACGCACAAGCAGCAGCTGGTGTTGCTGGCGTTGTTA
AGATGGTTCAGGCCCTCCGTCATGGTGTGATGCCGCGCTCTCTGTATGCGGACAACCCGACCACCAAGTTGAGTGGTCTTCCGGCCCGGTAGAACTGCTGGCTGAAGCAC
GTGAATGGGAGCGCGCAGACGGTCGCCCGCGTCGTGCAGGCGTTTCTGCCTTCGGTATCTCTGGTACTAACGCACACGTGATCCTGGAAGAGTTCGCTCCGGGTACTCCG
CACATCTCTGAGGGTGCTGAATCTGCTGTTCCGTGGGCGGACGTTGCAGTTCCGCTGGTGCTGAGCGGCAAAACCCCGGATGCGGTTCGCGGTCAGGCTCTGGCACTGCG
TGAACACCTGGCTTCTCACCCGGAGCTGCCATTACCGCACGTTGCGCGCGAACTGGCTACCTGCCGTACCCGTTTCGAGCACCGTGCAGTAGTTACTGGTGACCGTGAGG
CCGTGCGTGCAGGTCTGGACGACGTTAGCCCAGTTCTGGCTGGCTCTGGCCGCGTAGCGGCAGTGTTCTCTGGTCAGGGTGCGCAGCGTGCAGGCATGGGTCGTCAGCT
GGCGGACCATTTCCCGGTTTTTGACGAAGCTCTGAAGGAGGTATGTGGTCACCTCGACCCGGAACTGGGTCGCGGCCTGCGTGAAGTTATGTGGTCTGATGACCCGGAAC
AGCTGGGTCGTACCGAGTTCGCTCAGCCGGCTCTCTTCGCATTCGAGGTAGCACTGGCTCGTCTGTGGCAGTCTTGGGGCGTGTCTTTCACCGCAGTTGTTGGTCACTCCG
TAGGCGAAATCGCGGCCGCTGTGGTGGCAGGTGTACTGACCGTGCCGGATGCTGCGCGCCTCGTAGTTGCTCGTGGTCGCCTGATGCAGAGCCTGCCGGGCGGCGGTG
CCATGTTAGCAGTCGCTGCCGGTGAGGAGGAAGTTACCGCAACTCTGGGCGACTCTGCTCTGGTTGGTATCGCGGCTGTTAACGGCCCAGAAGCAGTTGTGGTTTCTGGT
GCTCGTGACGAAGTAGCTCGCGTTGGCGGCATCTGGCGTGAACGTGGTCGTCGTGTGGCTGAGCTGCGCGTTTCCCACGCTTTCCACTCTCCGCTCATGGAGCCAATCCT
GGACGAGTTTCGTGCAGTTGTAGAAAAGCTCCCGTTCCGTCCGCCAACTGTGCCGATCATCGCTACCGCAGACACCGCGCACCCGGTTGACACCGCTGCATATTGGGTAG
ATCACGCGCGTAACGCTGTACGTTTTGGCGACGCAATCGGTCGCATCCCGGACGCTGACCTGCTGATCGAAGTTGGCCCGGACGCAGCTCTGGCACCGCTGATCGAAACT
GGTCACACTGTACTGCCGTCTACCCGTCGCGGTCGTTCTGAAACCCACGCGGTGGTTACCGCTCTGGGTCAGGCACACGCACACGGTGCAGACGTGGACTGGGCGGCAC
TGCTCCCGCCGGCTCCACGCACCGATCTGCCGACCTACGCATTCCAGCGCCAGCGTTACTGGGACTCTGCTTCTGACGCCACTGCGCGTGTACGTGCAGGTGCTGACCCG
CGCCCGCACCCGATGCTGACCTCTCGTACTGACCTGCCGGGTGCCGGTGGTCTGCTGCTGTCTGGTCGCCTGACTCCGGGCTCTGACCCGTGGCTGTCTCATCACGTTGT
AATGGGCACCATGCTGCTCCCGGGCACCGGCTTCGTTGAGCTGGCACTGGAGGCTGCACGCTCTGCGGGTGCAGGTTCCGTTGATGAGCTGGTACTGCGCGCTCCAATG
GTGTTCACCGAAGGCCGTCCGCGTGACCTGCAAGTTTGGGTTGGTCCGGACCAGGGCGATGAACGCGAACTGCAAATTCGTACACGTGAGACTGGTGGTGACTGGACTCT
GCACGCCACTGGTCTGCTGGCTGCACGTACAGCAGACACTGAAGGTTTCCGTGATGGTGACTGGGCAGGTGAGGTATGGCCACCGGCTGGTGCCCAGCAGCTGGTCGGC
TCCTCCTTCTACGAAGAACTGGCTGCTCGCGGTTATGAATACGGCCCGGCATTCCACGGTACGAAGGCTCTGTGGGAGCGTGACGGTGACCTGTTTGCTGAGGTTGTACTG
CCGGAAGGTCAGCCACGTGGTTTCGGTATCCATCCGGCACTGCTGGACGCTGCACTGCACGCGCTCCCGATCACCGGCTCTCTGTACGAAGCAGGTGAAGTTCGCCTGCC
GTTCTCCTTCAACTCTGTGTCTCTGTTCTCCTCTGATGCACGCCGCCTGCGTGTACGCATTCGTGCTGAGGCTGACAGCGCAGCTGTTTGGATCACCGACGATGCGGGCTC
TCCGGTTCTGGCTATGGAGGGCCTGATCCTGCGCAGCATTGAACGCGCACAGCTGGAAGCAGCTGGTGCTACTGGGCGTACTGGTTGGTTCTCTGTGACCTGGCGCCAGC
TGGCACGCGCAGCTACCACCGACCGTGTTCCCGGTAACTGGCTGCTCCTGGGTGAAGTCCCGCCGGCTCTGGGTTCTCTGTTCGACGATCCGGTTACCGCAGCGTCTTGG
GATCGCTCTACTGCTCCGGACGGTGTTCTGGTGGGCGCTGGTCGTGCAGAAGACCTGCTGGCTGCTCTGCACGAAGTAGCTGGCCACCGTACCGGCCCAGTATGGTGCGT
AACCTCAGGTGCAGTAACTGTTGGTACTGACGACCCGGCAGCAGACGTACGTGCTGCCGGTGTTTGGGGTCTGGGTCGTGTAGCAGGCCTGGAACTGCCGGATCGTTGGG
GTGGCCTGGTTGACCTGCCGGAACGCATCGACGACGCTACCCGTCGCGCACTGGCTGGCATTCTGACTGGTGACGATGAAGGCGAAGGTGCTGACGACGCGGCAGAAGA
CCAGCTGGCGCTGCGTGACGGTTGCCTGTGGGCAC31GCCGTCTGGTAACTGCTTCTGCCCCGCAAGCCCGCACCTGGACCCCGAAAGGCACTGTTCTGATCACTGGTGG
CACGGGTGGCCTGGGTGCGCACGTTGCCCGCCGTATTGCGGAACAGGGCTCTGCTGATCGTATCCTCCTGCTGTCTCGCCAGGGTCCGGCAGCTCCGGGTGCGACCGAA
CTGCTGGCAGAAATCAGCGCTTTCGGCGCAACTGCTGAAGCCGTAGCTGCCGACGTGACCGACCGCGCAGCAATGGGCGGTCTGCTGGCAACTCTGGCAGCTGAAGGTG
CTCCGGTTCGCACCGTTGTACATGCAGCGGGCGTTGTTCGTGACGTGCGTATTGTTGAGACTGGTGCAGAAGCTCTGGCGGCTCAGATGGCAGCAAAAGTTGAAGGCGCA
CTGCTGCTGGATGAACTGCTGCCGGACCTGGACGATTTCGTTCTGTTCTCCAGCATCTCTGGTGTATGGGGTGCGGCTGGTCAGGCTGGTTATGCTGCCGGTAACGCTTGC
CTGGATGCGCTCGCTCGTCGCCGTCGTGAGCAGGGTCGCCCGGCTACCAGCGTTGCATGGGGTCCGTGGGCTGGCGGTGGTATGCTGACTGAACACGACGAACGTGAAC
TGCGTAAACGTGGTCTGACTCCGCTGCTGGTTCCGGCTGCTCTGCAAGCAATGGAACAGTCTATTATGGCAGACTCTGCGTGGGACCCGGTAGTGGCGGACATCACCTGGT
CTCGTTTCCTGCCGGCCTTCACCGTATCTCGTCCGTCTCCGCTGCTGGGCAGCTTCGAAGAAAAAACTGCACCGGGTCCGCTGGCTCCGTCTGATGGTCGTCGTACCGAAG
GCGAGGCCCAGTCTCTGACTCACCGTCTCGCAGCACTGCCGGACGCTGAACGTCTGCCGGTGCTGGCGGAAGTTGTGCGCACCCACGTTGCTGCTCTGATCGGCGAATCT
GGCCCGGAACGTGTTGGCCCTGATCGTGCTCTCAAGGAAATCGGTTTCGATAGCCTGATGTCTGTGGAGCTGCGCAACCGCTTCGCTGGTCTGATCGGTGTGAAACTGCC
GGCTACCCTGGTTTTCGACCATCCGACTCCGAACGCACTTGCTGTACACCTGCTGGGTCACCTGGACCTGGGCGCAGCTGGTACTGCCACCGCAGAGAAACCGCTGCTGG
AAGTTGTTGAACAGATCGAATCTCGCGTTTTATCTCCGCTGACCGACACATCCACTCGTAAGGCACTGGCTGGTCGCTTAACTGAACTGCTGGATCGTCTGGCTGCTCTGGA
TGCTCGTCCGGTTACTGGCACCGATGGTCTGGCCTCTGCAAGCGCAGCTGAACTGATGCAGTTCATCGACACTGAACTGGGTGACATCTCA

PnB (AccessionnumberAFJ05079): KS-AT 1 - DH-KR1 - ACP 1 - KS-AT 2 - KR 2 - ACP 2 - *linker*

ATGACCACCGATGAAAGCCAGCTGGTTGAATATCTGCGTAAAGTTACCACCGATCTGCAAAAAACCCGTAGCCGTCTGCGTGATGCAGAAACCAAACATCATGAACCGATTG
CCATTGTTGGTATTGGTTGTCGTTATCCGGGTGGTGTTCATGGTGCAGATGATCTGTGGCGTCTGGTTGTTGAAGGTCGTGATGCAATTAGCGGTTTTCCGCAGGATCGTGG
TTGGGATCTGGCAGGTCTGTATGATCCTGATCCGGATGCAGTTGGCACCAGCTATACCCGTGAAGGTGGTTTTCTGACCGGCATTGACCTGTTTGATGCAGATTTTTTTGGT
CTGAGTCCGCGTGAAGCACTGGCAATGGACCCGCAGCAGCGTCTGCTGCTGGAAACCAGCTGGGAAGCACTGGAAAGCGCAGGTATTCGTCCGGCAACCCTGCGTGGTA

GCCGTACAGGTGTTTTTACAGGTGTTATGCATCATGATTATGGTAGCCGTTTTAGCAAAGCACCGGATGGTTTTGAAGGTTATGTTCACATGGGTAGCGCAGGTAGCATGGC
AGTTGGTCGTGTTGCATATCATTTTGGTCTGGAAGGTCCGGCAGTTACCGTTGATACCGCATGTAGCAGCAGCCTGGTTAGCCTGCATCTGGCATGTCAGAGCCTGCGTACA
GGTGAATGTGATCTGGCACTGGCAGGCGGTGTTGCAATTATGGCAGGTCCGACCTTTTTTGTTGAATATAGCCGTCAGCGTGTTCTGAGCACCGATGGTCGTTGTCATAGCT
TTGCAGATGATGGTGATGGTACAGGTTGGAGCGAAGGTGTTGGTGTTCTGGCAGTTGAACGTCTGAGTGATGCACGTCGTCTGGGTCATGATGTTCTGGCCGTTGTTCGTG
GTAGCGCAGTTAATCAGGATGGTGCAAGCAATGGTATGACCGCACCGAGTGGTCCGGCACAGCAGCGTGTTATTGATAGCGCACTGCGTAATGCACGTCTGAGCGCAGAT
GAAATTGATGTTGTTGAAGCACATGGCACCGGCACCCGTCTGGGTGATCCGATTGAAGCACAGGCCCTGATGGCAACCTATGGTCGTGGTCGTAGCGCACAGCGTCCGCT
GTGGCTGGGTAGCCTGAAAAGCAATATTGGTCATACCCAGGCAGCAGCCGGTGTTGGTGGTGTGATTAAAATGGTTCAGGCACTGCGTCATGGTGTTCTGCCTCGTAGCCT
GTATGCAGAACGTCCGAGCACCGAAGTTGATTGGAGCGCAGGCACCGTTGCACTGCTGGCACGTGAACGTGCATGGGAACCTGTTGAAGATCGTCCGCGTCGTGCCGGTG
TTAGCAGCTTTGGTATGAGCGGCACCAATGCACATGTTATTGTTGAAGAATTTGTTCCGGAACCGGCAGGCGAAAGCGAAGCAGAACCGAGCGCACCGTGGGCAGAAAATA
GCGTTCCGCTGGTTCTGAGCGGTCGTACACCGGAAGCAGTTCGTGCACAGGCAGCAGCACTGCGTGATCATCTGGCAGCACGTCCGGAACTGAGCCTGGCACGTGCAGC
ACGTGAAAGCGCAGTTCATCGTACCGCATTTGAACATCGTGCAACCGTTACAGGTGATCGT32GATGCAGTTCTGGCAGGCCTGGGTCATGTTACTCCGGTTGCAGCCGGT
GGTGGTCGCGTTGCAGCAGTTTTTAGCGGTCAGGGTGCACAGCATGTTGGTATGGGTCGTCAGCTGGCAGCAGATTTTCCGGTTTTTGCCGGTGTTCTGGATGAAGTTTGT
GCAGTTGTTGATCCGCTGCTGGGTCGTAGCCTGCGTGAAGTTATGTGGTCAGGTAGCGCAGAACAGCTGGAACGTACCGAATTTGCACAGCCTGCACTGTTTGCATTTGAA
GTTGCACTGGCACGTCTGTGGCAGAGCTGGGGTGTTGAATTTAGCGTTCTGGCAGGTCATAGCGTTGGTGAAATTGCAGCAGCAGTTGTTGCCGGTGCACTGAGCCTGGAT
GATGCAGCCCGTCTGGCAGTTGTGCGTGGTCGTCTGATGCAGCGTCTGCCGGAAGGTGGTGCAATGCTGGCAGTTGCAGCGGGTGAAGAAGAAGTTGCAGCAACCCTGG
GCGATCCGGCACTGGTTGGTATTGCAGCAGTTAATGGTCCGGAAGCCGTTGTTGTTAGCGGTGCACGTGCCGAAGTTGAACGTGTTGCCGGTGTTTGGCGTGAACGTGGT
CGTCGTACCAGTCGTCTGCGTGTTAGCCATGCATTTCATAGTCCGCTGATGGAACCTGTGCTGGATGAATTTCGTGCCGTTGCAGATGAACTGGATCGTCGTGAACCGACCC
TGCCGGTTATGGCAAGCGCAGAAACCACCCATCCGTTTGCAACCGCAGCATATTGGGTTGATCATGCACGTCGTGCAGTTCGTTTTCATGATGCAGTTACCCGTCTGGCCGA
AGCAGATGTTGTTGTTGAAATTGGTCCGGATGCGGTTCTGGCACCGCTGATTGATACAGGTCGTACCGTTCTGAGCAGCTGTCGTCGTGAACAGAGCGAAACCCTGACCCT
GCTGACCGCACTGGGTGAAGCACATGCACATGGTGTTGAAGTGGATTGGACCACCCTGTTTCCGGCAGCACCGCGTGCCGATCTGCCTGCATATCCGTTTCAGCATCGTCG
TTATTGGCTGGCACCG

CCTGCACTGACAGGTGCCGGTGCAGATGCCCTGGACCATCCGCTGCTGAGCAGCCTGGTGGAACTGCCTGGTCAGGGTGGTGTTGTTCTGACAGGTCGTATTAGTCCGGA
TCGTGATCCGTGGCTGGCAGATCATGCAGTTAGCGGTGCCGTTCTGTTTCCGGGTACAGGTTTTCTGGAACTGGCACTGCGTGCCGCACGTCAGGCAGGTTGGCGTCAGG
TTGCCGATCTGGTTGTTCAGGCACCGCTGGTGCTGCCAGCAGCGGGTGCAGATATTCAGGTTTGGGTTGAACCGCCTGGTGAAGCAGAACGTGCACTGGTGGTTCGTGGT
CGTTCAGGTGATGGTGATTGGGTTGAATATGCAACAGGTCGTCTGGTTGAAGAACCGCCTGAAACCAGTCCGCAGGGTAGCCCTCCGACCGATGCAGAATGGGCAACAGG
TCAGTGGCCTCCGCAGGGTGCAGAAGAAATGCCGGTTGAAGAACTGTATGATGCACTGGCAGCCCGTGGTTATGGTTATGGTCCGGTTTTTCGTGGTCTGCGTGCACTGTG
GCGTAGCGGTGATGAACTGTATGCCGAAGTTGTTCTGCCGGAACGTGCACAGGATGGTCGTTATGATTGTCATCCGGCACTGCTGGATGCAGCACTGCATCCGCTGGCAGC
AGCAGCAGATAGTGGTCAGCAGGTTCGTCTGCCGTTTGCATTTGGTCGTGCCACCGTTCATAGTCCGGGTGCAAGCGAACTGCGTGTTCGTCTGCGTACGGGTGCAGAAAC
CATGCGTCTGGATGCAGTTAGCCCGACAGGTGAACCTGTTCTGACCATTGGTGAACTGGTTCTGCGTGCAGCAGATACCGCACGTCTGACAGCCCGTGCCGAAGGTCATGT
TCATCGTCTGGGTTATGAAGTTACCTGGCAGCTGCTGCCTGATCCGCCTCGTGCAGATCGTCTGCCTGGCACCTGGATTGTTCTGGCACCTCCGGCAACCGATGTTAGCTG
GACCCGTGATCTGGCAGAACATACCCTGGCAGTTGATCTGGATACCACACCGGATCGTCCGGGTCTGGTTGCACGTCTGGCAGATGTTACCCATGTTCCGACCGCAACCGC
ACCGGATGGCGTTCTGCTGTTTGCAGCACATCCTGATCTGCTGGTTACCGCACTGCAAGCACTGACCGATGCCGGTGTTGATGCTCCGGTTTGGTGTCTGACCCATGAAAG
TGATGATGAGCTGGATACCGCAGCAGTTTGGGGTGCAGGTCGTGCAGCAGCCCTGGAACTGCCGGATCGTTGGGGTGGTCTGGTTGATCTGCCTCCGGCAGGCGTTCGTC
CGGATCTGGGTTGTCTGGCTGGTCTGCTGAGTACCGATACAGGTGAAGATCATCTGCGTCTGCGTCCGGATGGTGTTAGCGTTCGTCGTCTGATTAAACCTGATCGTCCGA
CCCAGCCTCCGGCAGATTGGACCCCGAGCGGCACCGTTCTGATTACAGGTGGTACAGGTGCACTGGGTGGTCATGTTGCACGTTGGGTTGCAGGTCTGGGTGGTTGTAGC
CTGCTGCTGGTTAGCCGTCGTGGTCCTGAAGCACCGGGTGCACAAGAACTGCTGGCAGAACTGGCAGCAACCGGCACTCCGGTTCGTATTGTTGCAGCAGATGTTGCAGA
TCGTAGCGCAATGGCAGGTCTGGTTCTGGAAGCAGCAGATGCAGGCACCCCTGTTCGTGCAGTTTTTCATGCAGCGGGTGTTGCCGATGAAACACCTCTGCTGGAAACAAC
CCTGGGTCGTTTTCATGCCGTTCTGGAAGGTAAAGCCGGTGGTGCACGTGTGCTGGACGAAGTTCTGGGTGAAACCGATCTGGATGCATTTGTTGTTTTTAGCAGCGTTAGC
GGTGTTTGGGGTGGTGCAGGTCAGAGCGCCTATGGTGCAGGTAATGCAGTGCTGGATGCCCTGGCAAGCCGTCGTCGTGCACGTGGTCTGGCAGGCACCGCACTGGCAT
GGGGTCCGTGGTCAGGTGGTGGTATGGTTGAT33GATGATCTGGAACGTCGTCTGCGTCGTCAGGGTCTGAATCCGCTGCCGGTTGATGATGCCATTGCAGCACTGAGCAC
CGCAGTTCCGCTGGGTGAAAATCGTGTTCTGGCGGATGTTGCATGGTCACGTTTTCTGCCGCTGTTTGCCGCAACCCGTCCGGCACCGCTGTTTACAGCACTGCCTCCGAC
ACCGCGTCATACCGCAAGCGCAGCAACAGCAGCACCGGGTCCGACCGCAGAA

CGTCTGGCAGCACTGCCTGCCGCAGATCGTGCAAAAGCACTGAGCGATCTGGTTCGTGCAGAAATTGCAAGCGCAGTGGGTCATCATACCATGAGCGCAGTTGATGCCGA
ACGTCCGCTGGGCGAATTTGGTTTTGATAGCCTGATGAGCGTTCAGCTGCGTAATCGTCTGAGTGCAGCAACAGGTGTGCGTCTGCCTGCAACCCTGGTTTTTGATCATCCG
ACCGCAGGCGCACTGGCACGCCATCTGGAAAGCGAACTGGCAACCGAACCGAGCACCCGTCCGCGTCAGCCTGCC

ACCGCAGAAACCGCAGTTGCGGATGAACCGCTGGCGATTGTGGGTATGGCCTGTCGCTATCCTGGTGGCGTTGCAAGCCCTGAGGACCTGTGGCGTCTGGTGGCACAGG
GTCGTGATGCCGTTACCGCCTTTCCGACCGATCGCGGATGGGACCTGGCTGGCCTGTATCATCCGGATCGTAGCCGTGCAGGCACCACCTATGCAAGCGAAGGTGGCTTT
CTGGATGATCCGGCAGGTTTTGATGCCGATTTCTTCGGTATTTCACCGCGTGAGGCCCTGGCTATGGACCCTCAACAACGCCTGCTGCTGGAAGCAAGTTGGGCAGCAGTT
GAACATGCAGGTATTGCACCGGATACCCTGCGTGGCTCACGTACGGGTGTGTTTGTTGGCACCATGTATAATGATTATTTCAGCCGTCTGAGCAGCACACCGGAAAGCCTG
GAAGGTATTATTGGTATTGCAAATAGCAATAGCGTTATGAGCGGTCGCATTAGCTATCTGCTGGGTCTGCAAGGTCCTGCCGTGACCCTGGATACCGCCTGTAGCTCAAGCC
TGGTGGCACTGCACCTGGCAGGTCAGTCACTGCGTCGTGGCGAATGCGACCTGGCCCTGGCTGGGGGTGCCACCGTTATGGCAAGTCCGCATATTTTTGTGGAATTTAGT
CGCCAGGGTGGTCTGGCCGTTGATGGTCGTTGCAAAAGTTTTAGCGCAGATGCAGATGGCACCGGCTGGTCAGAAGGTGTGGGTCTGCTGGTGCTGGAACGTCTGTCAGA
TGCCCGTCGCCTGGGCCACGAAGTGCTGGCGGTTGTTCATGGTTCAGCCGTGAACCAGGATGGTGCCTCAAATGGCCTGACCGCACCGTCAGGTCCTGCCCAGCAGCGC
GTTGTGGAAGCAGCACTGGCACAGGCAGGTATTGCCGCAACCGAAGTGGATGCAGTTGAAGCGCATGGTACAGGTACACGCCTGGGCGACCCGATCGAAGCCCAAGCAC
TGATTGCCACGTATGGTCGTGAACGTGAAGCCGAACGTCCTCTGTATCTGGGTAGTCTGAAATCAAATATTGGCCACGCGCAGGCAGCCGCAGGCGTGGGTGGCGTTATCA
AAATGGTGCAAGCCCTGCGCCATGAACTGCTGCCTCGTACCCTGCACGCAGATACCCCGAGTCCGGAAGTAGATTGGAGCAGCGAAGCCGTTCGTCTGCTGACCGAAGAA
CGTCCGTGGCCTCGTGGTGATCGTCCTCGTCTGGCAGGCGTTAGCTCATTTGGTATTAGCGGTACAAACGCCCATGTTGTTCTGGCTGAAGGTGATCCGCTGGATGGTGAA
CCTGCCGAAGGTGCTCCGGTTAATGGTGAAAGCGTTGAAAGCGGCACCCCGATGGATACCAGCGATAGCGCAGCAGCCCGTGCACCGCTGCCGTATGTTCTGACCGCACG
TAGCGCAGCAGGTCTGCCTGCACAGGCACGTGCACTGCATAGCCATCTGATTGGTCGTCCTGGTCTGGAACCGGCAGAAGTTGCACGTAGCCTGGTTACCACCCGTAGCC
TGCATGATCATCGTGCGGTTGTTGTTGCAGAAGATCGTCAAGAGCTGCTGGACGCACTGGCAGCGCTGGCAGATCCGGCAACCACCACACCGCGTCGCACCGTTAGTACC
GGCACCGCAGGTCGTGGCCGTGTTGCGGGTCTGTTTTCAGGTCAGGGTGCCCAACGTCCGGGTATGGGTCGCGAACTGGCAGGTCGTTTTCCGGTGTTTGCAGGCGTGCT
GGACGAGGTTTGTGCCGTGGTGGACCCTCTGCTGGGACGTTCACTGCGCGAGGTGATGTGGTCAGCACCGGGTGAAGTTCTGGAACGCACAGAGTTTGCCCAGCCAGCC
CTGTTTGCCTTCGAGGTGGCTCTGGCTCGCCTGTGGCAGTCATGGGGAGTGGAATTTTCAGTGCTGGCAGGCCATTCAGTGGGTGAAATCGCAGCCGCATGTGTTAGCGG
TGTGCTGAGCCTGCCGGATGCCGCACGTCTGGTTGTGGCACGTGGACGCCTGATGCAGGCACTGCCTGAAGGCGGAGCAATGGTTGCGATTGCAGCAGGCGAAGATGAA
GTTAGCGCCAGCCTGGCCGATGTTCCGGATGTTGCCATTGCCGCTGTGAATGGTCCAGAAGCAGTGGTAGTTTCAGGCACCGAAGCCGATGTTCTGCGTAGCGCAGATCAT
TGGCGTGAACAGGGACGTCGCACCTCACGTCTGCGTGTGTCACATGCCTTTCATTCACCTCTGATGGAACCAATGCTGGACGATTTTGCAGCAGTTCTGACCGAACTGACCT
TTCATGAACCGGCACTGCCGATTAGCCCGAGCGCAGATAGCAGCCGTAGCTTTGCCAGCGCAGAATATTGGCTGGACCATGCCCGTCATGCCGTGCGTTTTGCAGATGCAC
TGAGTGGTCTGGATGGTGCGGATGTTCTGGTTGAACTGGGTCCTGATGCAGCCCTGGCACCTCTGGCAGGTACAGATAAACCTGTTCTGGTTTGTG34CACGTCGTAATCAG
CCGGAAGTTCGTACCCTGGTTACAGCACTGGGTGCCGGTCATGCGCATGGTGTGGCGGTTGATTGGACAGCAGTGCTGGGTGAAGGTCGTCAGGTGCGTCTGCCGACCTA
TAGCTTTCAGCATGAACGCTATTGGCTGGATGAAGATACCGCAGCCGGTCGTGGTACTGGTGCA

GCACATGATGCAGCGCAGGCACGTTTTTGGCAGGTTGTTGAAGATCAGGATCTGGATGGTCTGACCCGTACCCTGGGTCTGGATGCCGAAACCAGCCTGCGTGCAGCCCT
GCCGGTTCTGCATGATTGGCATCGTACCAGCAGCACCCTGGCGCAGGCAGCGGGTTGGCGCTATCGTGTTGCATGGGATCGTGTTCCGACCGATGATAGCGCAGTTGCAC
TGGATGGCACCTGGTGGATTGTTGTTCCGAGTGATGCAGCAGATGCCACCACCGCAGATGCAGTTCGTCGTGCCCTGGCAGCGGCAGGCGCAAATCCGCGTATTCTGACG
ATTGATCCGCATCGTACCGATCGTGCAGCACTGGCCAAAGAGCTGGCAGCCGCAGCCGATGGTACAGTTGCAGGTACAGTTAGCCTGCTGGCCGAAAGCGGTGGTGAAGA
TACAGGTCATCGTGGTGTGGCAGCCGGTGCCCTGGCCACCCTGGTTCTGCTGCAAGCGCTGCATGATGCAGATATTACCACCCGTCTGTGGACCCTGACCCGTGGTGCAG
TTCGTACAGGTCCGGCAGATACAGCTCCGGGTCCGTGGCACGCACAGGTTTGGGGTCTGGGTCGTGTTGCCGCACTGGAACATCCTGCACTGTGGGGTGGCCTGATCGAT
CTGCCAGCAGAAGGTGAACCGGCAGGTCTGGCAGCCGTTCTGAGTGGTACAGCCGGTGAAGATCAGGTTGCACTGCGTGAAGATGGTGTTCGTGCCCGTCGTCTGACACC
GGCAGATACCCATGATGCACCGGGTGCCGATGCACAGGCACCGGGTGCTGATCGTTGGACCGATGGTGCAGTTCTGATCACGGGTGGCACGGGTGCCCTGGGTGCACAT
ACCGCACGTATGCTGGCCAGCCGTGGTGCAAGCCGTCTGGTGCTGGTTTCACGTCGTGGTGCAGCCGCACCGGGTGCGGAAGCACTGCGTGCGGAACTGGAAACCCTGG
GTGCAACCGTTGATCTGACCGCATGTGATGTTACCGATCGTGATGCGGTTGGTCGTCTGGCAGAAGCCCTGGCAGCCGAAGGTACACCTGTTCGTGCCGTGGTTCATGCC
GCAGGCGTTGCAGCGGAACGTCCACTGACAGAACTGGTTGGTGATGATTTTTCAGCAGTTAGTGATGCAAAAGTTACCGCAGCAGAAATTCTGGATGATGTGCTGGGTGAT
GACCTGGCAGCCTTTGTTGTGTATAGCAGCATTGCAGGCACCTGGGGTTCAGCCGGTAATGGTCCGTATGCAGCCGCAAATGCACATCTGGATGCACTGGTTGAACGTCGC

CGTGCCCGTGGTGCCGTTGGCACCGCTATTGCCTGGGGTCCTTGGAGCGGTGGCGGTATGGCAGATGATCGTTTTCAAGAAGAAATGCTGCGTCGCGGTGTTAGCGCACT
GTCACCGCAGGGTGCCACCGCAGCACTGGCGCAAGCCCTGGAACATGATGATACCACCGTTACCGTTGTTGATGTTGATTGGGATCGTTTTGCACGTGTTTTTGCATGTAAT
CGTCCGAGTCCGCTGCTGCGTCATCTGACCGAACCTGCCGCAGCTGCCGGTGAAGCACCGGCACGTACCGAACTGGCAGAG

CGGCTGGCAGCCCTGGATGCAGGTACACGTCGTGGCGCAGTTCTGGATCTGGTGCGTGCGGAAGTTGCGGGTGTTCTGGGTCATGCAACCAGCCAGGCAGTTGCAGTTG
ATCGTGCATTTACCGATATGGGTTTTGATTCACTGATGGCCGTTGAACTGCGTGCACGTCTGGGTGCCGTTACAGGTCTGGCACTGCCGACCACCCTGGTGTTCGATCGTC
CGAGCCCTGCCGAAGTTGCAGGTTTTCTGTGTGATCGTTTTGAACCAGATCGTGATGCCCTGACACATGAAATTCTG

GAAAAACTGGATTGGCTGGAAAAAACGCTGCTGGATGCTGCGCATAGCCAGGGTGCCCGTGCACGTTTTGGTAGCCGTCTGGATGCGCTGCTGGCACGCCTGGATCGTAC
CACCGTTCCGCCTGAAAGTCGTCTGAGCGATCCGGATACCGATACCGATGCCATTGAAAGCGCAACCGCAGAGGAACTGCTGGCCTTTGTTGAAAAACACTTTGATTCA

PnC (AccessionnumberAFJ05066): KS-AT - KR - ACP - *linker*

ATGGTTATGACAGGCGAAGGTCGTAGCGATCAGCTGGTGACCGCACTGCGCAAGGTAACTCGTGACCTGCGTGACACCCAACGTCGCCTGCAAGTTGAAGAGGACCGTAA
CGGTGAGCCAATCGCTGTCGTTGGTATGAGCTGCCGTTTCCCGGGTCACGTCGACTCTCCGGAAGCACTGTGGGAACTGGTTTCTTCCGGCCGTGGTGCTTCTGGTTCTTT
CCCGGTTGATCGTGGTTGGGACCTGGACGCTCTGTTCTCCGAATCTGCTGACGGTAAAGTTCCGTCCTACGTTCGTGAAGGTGGCTTCCTGTATGATGCTGGTTGGTTCGA
CGCTTCTTTCTTCGGCATTTCTCCGCGTGAGGCGGTAACTATCGACCCGCAGCAACGTCTGCTGCTGGAAGTTGCTTGGGAATCCCTGGAACGTGCACGCATCAGCCCGCA
GGCACTGCGTGGTTCTGACGTAGGTGTTTTCGCTGGTGCAATGAACCAGGACTACGCAGTTCGCCTGCATGAAAGCATCGAAGATTTCGAAGGTTTCCTGACCACCGGCAA
CACCGGCTCTGTAGTTTCCGGTCGTCTGAGCTATACGCTGGGTCTGGTAGGCCCGGCTGTTACCGTAGACACTGCTTGTTCTTCCTCTCTGGTAACCGTGCACAT35GCAG
CACGCTCTCTGCGCTCTCGTGAATGCTCTCTGGCCCTCGCAGGCGGCGTAACCGTAATGTCTATGCCGACCACCTTTACTGAGTTCTGCCGTCAGCGCAACCTGGCGCCGG
ACGGTCGCGCTAAGTCCTTCGCAGCAGCAGCTGATGGTACTGCTTGGGCTGAAGGCGCAGGTATGCTGGTGCTGGAACGTTTAAGCGACGCTGAGCGTAACGGTCATCCG
GTGCTGGCTCTGATCCGTGGGTCTGCTGTAAACCAGGACGGTGCTTCTAACGGCCTGTCTGCTCCGAACGGCCCGTCTCAAGAACGTGTGATCTGGCAGGCCCTGCGCGA
TGCTCGTCTGACTGCTGACGAGATCGATGCAGTTGAAGCTCACGCAACCGGGACTCGTCTGGGTGACCCGATCGAAGCTCAAGCACTGCTGGCAACCTACGGTCAGGGCC
GTTCTGCTGACCGTCCGCTGTGGCTGGGTTCTCTGAAATCCAACATCGGTCACGCACAGGCAGCTGCTGGTGTGGGCGGTGTTATCAAGATGGTAATGGCTTTACGCCACG
GCGTTCTGCCGCAGACCCTGCACGTGGACGCGCCGACCCCGGAAGTTGATTGGTCTGCTGGTGAGGTTCGCCTGCTGACGGAGCGTCGTGAATGGCCGCGTGCAGGCCG
CCCGCGTCGTGCTGGCGTATCCTCTTTTGGTGTGTCCGGTACTAACGCTCACCTGATCCTGGAAGAGGCGCCGGCAGCTCCGCCTCGTGACGGTGCAGCTGACCGTGCTG
ACGATGGTGGTGGCGCTACCCTGCCGTGGGTTCTCTCTGCACGTACCGCAGCTGCTGTGCGTGAGCAGGCTCGCCGTCTTCATGGTCACCTGACTGACCATCCGGAGCTT
GTTCCGGCACAGGTTGCTCGTTCTCTGGTAACCACTCGTTCCACCTTCGAACAGCGCGCAGTTGTTCTGGGTACTGACCGCGCTGAGCTGCTGGACGGTCTGGATTCTATC
GTTCAGGGTGCCCCGGACGCACGTACTGTTACCGGCTCCGCTGTTGGTGGTCGTGAGGTAGTATTCGTGTTCCCAGGGCAGGGTGGTCAGTGGGCTGGCATGGCACTGG
AACTGATGGAAGAATTTCCGGTTTTCGCTGAAACCCTGCACTCTTGCGCTGACGCCCTGGCTGACTACGTTGACTGGTCTCTGCTGGACGTACTGCGCGAGGCAGAGGGTG
CTCCGGGTCTGGACCGTGTTGACGTCATCCAGCCGGTTCTGTTCTCTGTAACCGTTGCACTGGCAGACCTGTGGCGTAGCCTGGGCATCGAGCCGTCTGCTGTCGTTGGTT
CTAGTCTGGGCGAGATCGCAGCTGCATACACTGCTGGTACTCTGAGTCTGGCAGACGCGACCAAAGTGGCCGTTCTGCGCAGCCGTGCACTGCTGGAACTGTCTGGTCGT
TCTGGCATGGTTTCCGTGCCGCTGGGTCGTGCTCAGGTAGAAGAACTGATCGCAGCTTGGCCGGATCGCCTGTTCGTGAGCGGTGTTAACTCTCCATCTATCTCCGTAGTT
TCTGGCGACAACGAGGCAGTGGACGAACTGCTGGCGGTTTGTGCTGAGCGTGGTGTTCGTGCACGTCGTGTTGCTACCGACTGTGCTTCTCACTACCCGGCTGTAGAAGC
TCTGGAAGAGCGTCTGCTGACGGACCTGGCAGACCTGGCTCCGACTCCGGGTCGTATCGCTTTCCTGTCCACCGTTTCTGGTGAAAGCGCAGAAACTGAACCAGTACCGG
ATGCTGCTTACTGGTATCGTAACACTCGCCGCACCGTTGAGTTCGAGCCGGTTATTCAGCGTCTGGCAGCGACTGGTTCCAAAGTTTACATCGAAATCTCTCCACATCCGGT
GCTGCAAGTTGCTCTGAGCGAGATCGTGGAAGGTGAATCTCGTGAAGCTGCGGTTCTGAGCACTCTGCGTCGTAACACTTCTGACCGCCGCGCTTTCCTGACCAGCCTGGC
GAAAGCATACGTTTCTGGTGTAACTGTGGACTGGGCCGCACTGCCGTCCCTGGCGGGCGCAGCACAGGTGGACCTGCCGACTTATGCATTCCAACGTGAGCGCTACTGGC
CGCGCCCGGCAGCTGCAAGTAACGGCGGTCACGGT

GCTGGTACAGGTGCTCGTGCTGGTGTTGGCCACGGTACTGTTGACGCCCACTTCTGGGAAGCTGTAGAGAACGGCGACCTGGGTAGCCTGGGTCCAGACGTACGCTTTGA
CGACGAAACCCCGCTGAAGGAGGCACTGCCGGAACTGGCTTCTTGGCACCGTCACGGTCTTGAACAGGCACGCGTTGACGGTTGGCGTTACGTTGAACGTTGGCGTCCAC
TGGACGTTCCGGCTACTGGTCCACACGGCAAATGGCTGCTGGTGCACCCGGGTGGTACTGAATCCGATGCGACGACGGAATGGGTACGCGAATCTCTGCTCGAGGCTGGT
TGTTCTGTAGTGGACCTGCTGGTTGACACTACTGACACTGACCCGGCAGCTGTGACGGAGCGTCTGAACCAGGCTTGCGCTGGTGGTGGCCCGGAGCCGGTTGGTGTGGT
TAGTCTGCTGGCGTTCGACGGCCGCCACGACACTGCTCGCCCGTCCGTACCGCGTGGTACAGCAGCTACTCTGGCTCTGGTACGTGCACTGGGTGCAGCGGGTATCACTG
CTCCGCTGTGGTGCCTGACCCAGGGTGCAGTTACAACTGGCACGGGTGACCGTCTGGACGCTGTAGAACAGGCACAAATCTGGGGTCTGGGCCGTGTGGTTGCACTGGAA
CACCCGGATCGTTGGGGCGGTCTGGTTGACCTGCCGTCTACACTGGACCCGCACGTACGTACTCAGCTGTGTGCAGCACTGGGCGGTGCTCATACCGACGAGGACCAGCT
GGCCCTGCGTCCGGTGGGCATCCTGGGTCGCCGTCTGGTTCCGGATGACGGTGACCGCCAGGCAGCTCGTACTGCACCGTGGCGTCCAGCTGGTACTGTTCTGATCACA
GGTGGTACTGGTGCTCTGGGCGCTCAC36GTTGCGCGCTGGGCCGCTGCTCAGGGTGTAGGTCACCTGCTGCTGGCTGGTCGTCGCGGTGCGGATGCACCGGGCGTTGC
AGAACTGCGTGCAGACCTGGAGGCATCTGGTGCAACTGTAACCGTAGCTTCTTGCGATGTGGCAGACCGCCGTGCTGTTCAGGAACTGCTGGATGGTATCCCGGATCGCC
ACCCGCTGACCGCCGTGTTCCATGCAGCTGGCGTCCTGGACGATGGTATGACGGCTGATCTGGACACCGCACGTCTGGAGACCGTACTGGCCCCTAAAACCGAAGGTGCT
CGTCTGCTGGACGAACTCACTCGTGATACCGAACTGAGCGCATTCGTACTGTTCTCTGGTTTCGCAGCAACCGTGGGTTCTAGTGGTCAGGCCGGCTACGCGGCAGCTAAC
GCTCACCTGGACGCACTCGCGCAGCGCCGTCGTGCTGACGGTCTGCCGGCAACTTCTATTGCGTGGGTTCCGTGGACTGGCGGCGGTCTGGTTGACGAAGGTATCGAAG
AGCGCCTGCTGCGTCGTGGTCTGAGTGCTATGGAACCGCGCCTGGCAGTGCTGGGTCTGTCTGCCCTGCTGAGCGGTGGCCCGGATGGTCCGGCTAACGTAGCACTGGT
AGACGTTGATTGGCGTCGTTTCCTGCCGTCTTTCACCGCTTCCCGCCCGTCTCCGCTGCTGCGTGAGCTGCACCAGCCGTCTCCGGCGGACCCGGGTGCTACTGCTGGTC
GTGACGGCACCCCAGGCGATGGCGCAGGTCGTACTCTGGCAGAACGT

CTGCCGGGTCTGACCGACGCTGAACGTGACCGCCTGCTGCTTACAACTGTTCGCGCACAGGTGGCTTCCACTCTGGGTTATCCGGGCCCGGATGCTGTACCGCCGCGTCA
GCAGTTCAAAGAACTGGGTATCGACTCTCTGACTGCATTAGAACTGCGCAACGGTCTGCAAGCTGTTGCGGGCGTAGCACTGCCGGCTACTCTGGTTTTCGATCATCCGAC
CCCGGAGGCAGTTGCTGGTCACCTGTCCCGTACTCTGCTGGGTGATGGTGCACACACC

AGCTCTTCCCTGTTTACCGAAATCGACCGCCTGGGCTCCGCACTGGGTTCCGCTGACCTGGATGAAGCAGAACGTAACCTGGTAGCAACTCGTCTGCGTTCCCTGGCTTCT
CGCTGGGATGCTGGTCCGTCTTCTGGCGCTGGTGACGTTGCACAGACTCTGGCTGACGCAGACCGCGCGGAGGTACTGGCATTCATCGATCGTGAACTGGGCATCTCTTC
A

PnD(AccessionnumberAFJ05067): KS-AT - DH-KR - ACP - *linker*

ATGCGTTCCGAAGAACAGCTGCTGGACTACCTGAAACGTGTAACTGCTGACCTGCACGACACCCGTGTGCGTCTGCAAGCTACCGAACAGGCCTCTCGCGAACCAATCGCA
ATCGTTGGCATGGGTTGTCGTTACCCAGGCGGTGTTTTCTCTCCGGAGGACCTGTGGCAGCTGGTTGCAGAAGGCCGCGACGTAATCGGTGACCTGCCGACTGATCGTTCT
TGGAACCTGGACACTCTGTACGATCCGGACCCGGCTGCACCGGGCACGACTTACGCTCGTGGTGGTGGTTTCGTTGCTACCGCGACCCACTTCGACGCAGGCTTCTTCGG
CATCTCCCCTCGTGAAGCAGCTGCAATGGACCCGCAGCAGCGTCTGGTACTGGAGACTGGTTGGGAAGCTCTGGAGCGCGCGGGCATCCCGCCGACCTCTCTGGGTGGT
TCTGATACTGGCGTTTACATCGGTACTGGTATGCAAGACCACATCATCCACCTGCAAAAAGCTCGCCAGGAGGCTGAAGGTTTCGTAGGCACGGGTAACGCGATCTCTGTG
GTTTCTGGTCGTCTGGCTTACACCCTGGGTCTGGAAGGCCCGGCTGTTTCCGTAGACACGGCCTGCTCTTCTTCTCTGGTTGCACTGCACCTGGCTGTTCAGGCTCTGCGT
GCTGGTGAATGCTCCCTGGCTCTGGCAGGGGGTGCTACGGTGATCAACACTCCGGTAATGTTCGTTGAGTTCTCTCGTCAGCGCGGTCTGTCCCCGGACGGTCGTTGCCG
CTCTTTCGCTGCTGATGCTGATGGCACGGGTTGGAGTGAAGGCGCGGGCGTACTGGTTCTGGAGAAACTGAGCGACGCTCGCCGTCACGGTCATCAGGTTCTGGCAGTTG
TTCGTGGTTCCGCTGTTAACCAGGACGGTGCTTCTAACGGTCTGACTGCTCCGAACGGCCCGTCTCAGCAGCGCGTTATCCGTCGTGCTCTTACCGCAGCTGGCCTGACCG
CAGATGAAGTCGACGTAGTAGAGGCACATGGTACTGGCACCCGTCTCGGGGACCCGATCGAAGCTCAGGCTCTGCTTGCAACCTACGGCCAGGGCCGTTCCGCTGACCGT
CCGCTGTGGCTGGGCTCTCTGAAGTCTAACATCGGTCACGCTCAGGCAGCAGCTGGCGTGGGTGGTGTTATCAAATCTGTAATGGCACTGCGTCACGGTGTTCTCCCGCAG
ACTCTGCACGTTCACGCACCAACCCCGGAGGTAGACTGGTCTGCTGGCGAAGTGCGCCTGCTTACCGAGCGTCGTGACTGGCCACGTGCTGGTCGTCCGCGTCGTGCAG
GTGTATCCAGCTTCGGTATCTCTGGCACTAACGCTCACCTGATCCTGGAGGAAGCACCAGATGAAGCGCCGGAAGAAGCTCCAGACGACGCAACCGACCGCGCACCGCGT

TTGCGTGGTATGCCGTGGGTCGTGAGCGGTCGTGGTCGTCAGGCTCTGCGTGCGCAGGCTGCGCGTCTGCGTGACTGGGCAGCTGGTCACCCGGATGTTGCACAGGAAG
AAGTGGGTCTGGCTCTCGCGACCCGCCGTTCTGTATTCGAGCACACCGCTGTGATCACCGGCGCAGACCATGATGAACTGCTGAACGGTTTATCTGCTCTGTCTGAAGATC
GCCCGGCACCGGGTGTTCGTCTGGAGACAGCTGCTGGTGGTGGCCTCGCATTCGCGTTTACTG37GTCAGGGTGCACAGCGTCCGGGTATGGGCCGTGGTCTGTATGAGA
CGTTCCCGGCTTACGCGGAAGCATTCGACGAGGCTTGCGCTGCACTGGACCCGCACCTGGAACGCCCGCTGGCTTCCGTTGTGTTTGCAGACGGCCCGGCTGACGCTGA
GGCGCTGCAAACCACCGCTTATGCCCAACCGGCTCTGTTCGCAGTTGAGACCGCTCTGTTCCACCTGATGCAGTCTATGGGTGTTCACCCGGACCTGCTGATCGGCCACTC
TGTTGGCGAACTGAGCGCTGCGCACGCAGCTGGCGTTCTGAGCCTCCAAGATGCTGCACGCCTGGTAGCTGCTCGTGGTCGCCTCATGCAATCTCTGCCGGAGGACGGTC
GTATGCTGGCCATCCAGGCATCTGAAGACGAGGTTCTTCCGTCCGTAGCTGAACTGAGCGAGAAGGCTGGTGGCGTTGCAGTTGCTGCTGTAAACGGCCCGGCATCTGTA
GTTGTATCTGGTCGTACTGAGGCTGTTCATACTCTCGAAAAAGAATTTGCAGGTCGCGGTCGTCGCGTACGTTACCTGGACGTGTCTCACGCATTCCACTCCCCACTGATGG
ACCCGGTTCTGGACGAGTTCGCACGCATCGCGGCTTCTGTTACCTTCCGTCCGGCTACCACCCCGGTTATCTCTAACGTGACTGGTGACCTGATCGGTGATGACCGTCTGG
CTGACCCGTCCTACTGGGCCGATCACATCCGTGCTACCGTTCGTTTCGCAGACGGTGTGCGTGCTCTGGCACGTGAACAGGTTGACACCGTAGTTGAACTGGGCCCGGAC
GCTGCACTGACCGCTCTTTGTGGTGAAATCCTGGACCAGGACACCGCAGCTTTCGTACCGACTCTGAGCCGTAAACACGACGAAACCTCTACTTTCCTGACTGCTATGGCTC
GACTGCACGCTCGTGGCATTCCGGTTCGTTGGCCGGCTGCGACCACTCCGTCTGTAGAAAGTGCTGATCTGCCGACCTACGCTTTCCAGCGTGAACGTCATTGGCTGGAC
GGTGTAGCGGAAACTGACGTGGCCGGCACTGGTCTGACC

GGCATCGGTCACCCGCTGCTCCCGGCTGAAACTGCACTGCCGGGTACTGAGGGTGTTGTGCTGACTGGCTCTCTGAGTCTGCACGACCATGCTTGGCTGGCAGATCACGC
TGTTCTGGGCGTAGTACTGGTGCCGGGAGCAGGTCTGCTGGACATGGCGCTGACAGCAGCTGAACATGCAGGCTGCACCCAGGTTGAAGAGCTGACCCTTGAATCCCCGC
TGATTTTACCGGAGGTTGGTGCTCGTAGCGTACAGGTACTGGTAGGTGCGGCTCAGGACTCCGGGCAGCGTGCCATCACCATCCACTCCCGTCCGCAGGACGGTGACCCG
CACGCAGCATGGACCCGCCACGCCACCGGCCTGCTGGCTACTGGTCCACAGGAAGAACCGGCTACCTTCTCTGAAGCATGGCCGCCGACTGGCGCTGTACCAGTTCCGG
TTGACGACCTGTACCTCCGCCTGACTGAAGGTGGTGTGGATTATGGTCCTTCTTTTCGTGGTCTGCGCGCAGCCTGGCGTCTGGGTGAAGACTTCTACGCAGACATCGACC
TGCCGCACCTGTCTGACGTAGAACGTTTCACCCTGCACCCGGCACTGTTAGACGCGGCTCTGCATTCTCTGGCACTGCCGGGTGCTATCCTCCATACCGGCCAGGCACACC
TGCCGTTCTCTTGGTCTGGTGTGCGTCTGCACGCCTCTGGTGCAGACGCCCTGCGTATCCGCGTTCGTGGCACGGGTTCTTCCTCAGTATCTCTGGAACTGGCAGACGGTA
CTGGCCGCCCGGTAGCAACTGTTGGCGAGCTGGCTCTGCGTCCGGTATCTCAGGAACAACTGCGTACCCCGGGCGCAGATCCGACTAGCCTGTACACCGTAGAGTGGCC
GGTAAAGGAACTGCCGGAACGTGCTGAAGGTTCTGCAACACGTGCGTGGGCAGTAATCGGTGCACCGGAGCCAGCTGGTTACGCGGTTGAAGGTGTTGAGCTGTCTCACT
ATGCTTCTCCGGCTACCCTGGCAGCAGCTCTGGATGCTACCGACGTGCCGGTTCCGGAGGCTGTTCTGGTGCCGTGCTTCTCTCCACACTCCCCGGGTTGTCCACCGGGT
GCATCTGCTGGCCTGGTTGAGCGTGCGCACGACTATGCTGCAACTGTACTGGACCTGGTACGCACCTGGCTGGGTGATGCTCGTTTCGAGGCCTCTCGCCTGCTGCTGCT
CACCCGTGGCGCAGTTACCCCAGACGCAGGCCACCACCCGCTGTCTGACGAGCTGGGCTCCCTGGCATGGGGTCTGGTCCGCACAGTACAGAACGAACACCCGGGTCGC
GTCCTGGTAGCAGACCTGGATGAAGATCCGGCTTCTTGGTCTGTTCTGCCCGGCGTGCTGGCACATGAGGAACCGCAGGTTGCTGTTCGTCGTGGTGTAGCGCACGTACC
ACGCCTGACTGCTGCACGCGCAGCAGCGGAACGTGCAACCCCGTTCGACTCCGTGGGCACCGTACTGGTTACTGGGGGTACTGGTGGCCTGGGTTCTCTGCTGGCTCGC
CACCTGGTAGTAGAGCACGGTGTACGCCATCTGCTGCTGACTTCTCGTCGCGGCCCGCAGGCTCCGGGTGCAGCTGCTCTGGCAGCTGAACTGACTGAACTGGGTGCACA
GGTTACCGTTACCGCTTGCGACATGGCTGACTCCCCGGCGGTTGAAGAACTGCTGGGTAGCCTGCCGGCTGGCCACCCGCTGACCGCAGTAATCCACACCGCTGGTGTGC
TGGATGACGGTCTGGTTCAAGACCTGACCCCGGAACGTCTGAACACCGTGCTGCGCCCGAAAGCTGATGCGGCTGTTGTTCTTGACCGCCTGACCCGTCACCTGGACCTG
GCAGCTTTCGTCCTGTACTCTAGCGTAGGTGGCACCCTCGGTGGTCCGGGCCAGGGCAACTACGCCGCAGCTAACGCATTCCTGGACGCACTTGCTCAGC38GTCGCCGC
GCCGAGGGCCTGCCGGGTCTGTCTCTGGGTTGGGGTCTTTGGTCCGACACCACTGGTATGGCGGCTGAGATCGGTACTACCCATGTGGGCCGTCTGAACCGTTCTGGTCT
GGTTACCATGTCTCCGGCTGAAGGTCTGGCACTGTTCGATGCTGCAATCTCCGGTGGCTATGGTCCGGTTGTCCTGCCTGTACGCCTGGACCTGCCGGCGCTGAAGGCAC
GCGCTACTTCTGGCGCTCTGCCGTCCGTTCTGGCAGACCTGGTACGTACCCCGGCGGGTCCGGCACGCGCGGCAAAATCTGGTACTGTGTCTGGTGCTGCTCTGCGTGCA
GCT

CTGTCTCAGCTGTCCGAAGACGAGCGCCGTCGTATGCTGCTCGACGTTGTACGTGAAAACGTTGCGGCTGTTTTGGGCCTGCGTCAGGACGGCGCTATGGACGAGGAGCA
GCCGTTCAAAGACCTGGGCCTGGACTCTCTGACGGCAGTAGAACTGCGCAACCGTCTGGCCAGCGCTACTGGCCTGCAACTGCCGGCAACCCTGCTGTTCGACCTGCCTT
CTCCGAGCGCTCTGGCTCACCACCTGCTGACTCGCATGGGTTCTCAAGAGAGTAAATCTCCGGTAGCTGAAGCAGTTGACCATCTGACCGCGCTGCTG

ACCACTCACGACGTAGGCGACCTGGAGCGTTCCCAGGTTACCGCCCGTCTGCGTTCTCTGCTGTGGCGCCTGGACGACGGTGGCGTAGAGTCTACTGAAGACGCGGCCG
ACCAGGCAGAAACTGACGACGACATCTTCGCACTGGTTGACCGTGAACTGGGTCTGGCTTCA

DEBS thioesterase (1MO2_A): used to replace the *linker* sequences in case of PnB_{DEBS}, PnC_{DEBS} or PnD_{DEBS}

AGCGGGACTCCCGCCCGGGAAGCGAGCAGCGCTCTTCGCGACGGCTACCGGCAGGCGGGCGTGTCGGGCAGGGTCCGGTCCTACCTCGACCTGCTGGCGGGGCTGTC
GGACTTCCGCGAGCACTTCGACGGCTCCGACGGGTTCTCCCTCGATCTCGTGGACATGGCCGACGGTCCCGGAGAGGTCACGGTGATCTGCTGCGCGGGAACGGCGGCG
ATCTCCGGTCCGCACGAGTTCACCCGGCTCGCCGGGGCGCTGCGCGGAATCGCTCCGGTTCGGGCCGTGCCCCAGCCCGGCTACGAGGAGGGCGAACCTCTGCCGTCG
TCGATGGCGGCGGTGGCGGCGGTGCAGGCCGATGCGGTCATCAGGACACAGGGGGACAAGCCGTTCGTGGTGGCCGGTCACTCCGCGGGGGCACTGATGGCCTACGC
GCTGGCGACCGAACTGCTCGATCGCGGGCACCCGCCACGCGGTGTCGTCCTGATCGACGTCTACCCGCCCGGTCACCAGGACGCGATGAACGCCTGGCTGGAGGAGCT
GACCGCCACGCTGTTCGACCGCGAGACGGTGCGGATGGACGACACCAGGCTCACCGCCCTGGGCGCCTACGACCGCCTCACCGGTCAGTGGCGACCCCGGGAAACCGG
GCTGCCGACGCTGCTGGTCAGCGCCGGCGAGCCGATGGGTCCGTGGCCCGACGACAGCTGGAAGCCGACGTGGCCCTTCGAGCACGACACCGTCGCCGTCCCCGGCGA
CCACTTCACGATGGTGCAGGAACACGCCGACGCGATCGCGCGGCACATCGACGCCTGGCTGGGCGGAGGGAATTCGAGCTCCGTCGAC

Npt (OSY40025):

ATGATTGAGAAGTTACTCCCGGCGCCAGTCAGAACGGCAGAGACTTTCGACGATGCGCCTTTATCTGAAATGTTCCCCGAAGAGTGGGCGCAGGTTGCAAACGCTGTACCC
AAACGCCAACGTGAGTTCGGTACTGTACGAGGGTGCGCTCGTCGTGCCCTGGCCGAGCTTGGCTTCGCTCCGGCACCATTGCTGCCTGGACCTCATCGTGAGCCGCAGTG
GCCAGATGGGGTTGTGGGCGCGATGACGCACTGCGCGGGATATCGCGCTGTAGCGGTGGCACGCGCCGGCGAAGTTCGCACAATCGGCCTGGATGCCGAACCGAATCTC
CCACTAAATGACCCGGGCGTTCTTGACCTGGTGACATTACCGGAAGAACGGGACCAGATCCGGCGCCTCGCCGCCCTTCAACCGGAAGTCTGTTGGGATCGCTTGGTCTTT
TCCGCAAAAGAAAGTGTCTACAAAGCCTGGTTTCCGCTGACGCGCCGTTGGTTGGATTTTGAAGAAGCACTGCTGACCTTTGATCCGACCAACGCGACCTTTACCGCGCAG
CTGCTGGTGCCGGGCCCGGTGGTTGATGGTCGTGAACTGACCGAATTTTCGGGTCGTTGGCTGGTGGGTAGCGGTCTGGTCGTTACCGCGATTGTGGAAATGGTGTCA

References

1. Fushimi, S.; Furihata, K.; Seto, H., Studies on new phosphate ester antifungal antibiotics phoslactomycins. II. Structure elucidation of phoslactomycins A to F. *The Journal of antibiotics* **1989,** *42* (7), 1026-36.

2. Chen, Y. L.; Zhao, J.; Liu, W.; Gao, J. F.; Tao, L. M.; Pan, H. X.; Tang, G. L., Identification of phoslactomycin biosynthetic gene clusters from Streptomyces platensis SAM-0654 and characterization of PnR1 and PnR2 as positive transcriptional regulators. *Gene* **2012,** *509* (2), 195-200.

3. Vogeli, B.; Geyer, K.; Gerlinger, P. D.; Benkstein, S.; Cortina, N. S.; Erb, T. J., Combining Promiscuous Acyl-CoA Oxidase and Enoyl-CoA Carboxylase/Reductases for Atypical Polyketide Extender Unit Biosynthesis. *Cell Chem Biol* **2018,** *25* (7), 833-839.e4.

4. Geyer, K.; Sundaram, S.; Susnik, P.; Koert, U.; Erb, T. J., Understanding Substrate Selectivity of Phoslactomycin Polyketide Synthase by Using Reconstituted in Vitro Systems. *Chembiochem : a European journal of chemical biology* **2020**.
